# Supplementary material for: Factors related to mortality in patients with acute respiratory distress syndrome (ARDS) in a lower middle-income country: A retrospective observational study
Source: PLoS One. 2025 Nov 18;20(11):e0337071. doi: 10.1371/journal.pone.0337071 (PMC12626306; doi:10.1371/journal.pone.0337071)
Supplement: S1 File — (PDF) [file pone.0337071.s001.pdf]

SUPPLEMENTARY RESULTS

ARTICLE TITLE

Factors related to mortality in patients with acute respiratory distress syndrome (ARDS) in a lower middle-income country: a retrospective observational study

AUTHORS

Co Xuan Dao, Chinh Quoc Luong, Toshie Manabe, My Ha Nguyen, Dung Thi Pham, Quynh Thi Pham, Tai Thien Vu, Hau Thi Truong, Dai Quoc Khuong, Hien Duy Dang, Tuan Anh Nguyen, Thach The Pham, Giang Thi Huong Bui, Cuong Van Bui, Quan Huu Nguyen, Thong Huu Tran, Tan Cong Nguyen, Khoi Hong Vo, Lan Tuong Vu, Nga Thu Phan, Loc The Vu, Cuong Duy Nguyen, Thom Thi Vu, Anh Dat Nguyen, Chi Van Nguyen, Tuan Quoc Dang, Binh Gia Nguyen, Son Ngoc Do

TABLE OF CONTENTS

S1 Table. Prior hospitalization, inter-hospital care, complications, and outcomes in patients with acute respiratory distress syndrome, according to the type of inter-hospital transportation

S2 Table. Baseline characteristics of patients with acute respiratory distress syndrome, according to hospital survivability

S3 Table. Respiratory viral pathogens in patients with acute respiratory distress syndrome, according to hospital survivability

S4 Table. The clinical and laboratory characteristics of patients with acute respiratory distress syndrome upon admission, according to hospital survivability

S5 Table. Initial chest imaging findings of patients with acute respiratory distress syndrome, according to hospital survivability

S6 Table. Comparative analysis of prior hospitalization and inter-hospital care in patients with acute respiratory distress syndrome before and during the COVID-19 pandemic

S7 Table. Comparative analysis of demographics and comorbidities in patients with acute respiratory distress syndrome before and during the COVID-19 pandemic

S8 Table. Comparative analysis of respiratory viral pathogens in patients with acute respiratory distress syndrome before and during the COVID-19 pandemic

S9 Table. Comparative analysis of clinical and laboratory characteristics in patients with acute respiratory distress syndrome upon admission before and during the COVID-19 pandemic

S10 Table. Comparative analysis of arterial blood gas parameters, chest x-ray findings, and severity of illness upon admission in patients with acute respiratory distress syndrome pre- and during the COVID-19 pandemic

S11 Table. Comparative analysis of management strategies for patients with acute respiratory distress syndrome before and during the COVID-19 pandemic

S12 Table. Comparative analysis of clinical time course and complications in patients with acute respiratory distress syndrome pre- and during the COVID-19 pandemic

S13 Table. Overall diagnostic performance of the Charlson Comorbidity Index, PaO2/FiO2 ratio, and Sequential Organ Failure Assessment Score upon admission for predicting hospital mortality in patients with acute respiratory distress syndrome

S14 Table. Factors associated with hospital mortality in patients with acute respiratory distress syndrome

S15 Table. Management strategies for patients with acute respiratory distress syndrome, according to the severity of illness upon admission

S16 Table. Management strategies for patients with acute respiratory distress syndrome, according to the severity of hypoxemia upon admission

S17 Table. Breakdown of missing data

S1 Table. Prior hospitalization, inter-hospital care, complications, and outcomes in patients with acute respiratory distress syndrome, according to the type of inter-hospital transportation

Variables	Hospital ambulances (n=63)	EMS (n=117)	Private ambulances (n=30)	Public or own/private transport (n=4)	Missing data on the type of inter-hospital transportation (n=139)
**Prior hospitalization**					
Prior hospitalization, no. (%)	63 (100.0)	96 (82.1)	30 (100.0)	0 (0.0)	127 (91.4)
Duration of stay (day), mean (SD), n=313	2.56 (1.68)	4.73 (5.08)	3.60 (3.48)	NA	1.05 (0.22)
MV applied[a], no. (%)	45 (71.4)	66 (68.8)	18 (60.0)	NA	124 (99.2)
Duration of MV (day), mean (SD), n=252	1.59 (1.26)	1.79 (2.03)	2.33 (2.83)	NA	1.10 (0.30)
**Inter-hospital care**					
Inter-hospital care provider, n=201					
Bystanders[b], no. (%)	8 (14.3)	10 (8.5)	19 (76.0)	2 (100.0)	1 (100.0)
EMS staff, no. (%)	0 (0.0)	105 (89.7)	1 (4.0)	0 (0.0)	0 (0.0)
Hospital doctors, no. (%)	1 (1.8)	0 (0.0)	0 (0.0)	0 (0.0)	0 (0.0)
Hospital nurses, no. (%)	47 (83.9)	2 (1.7)	5 (20.0)	0 (0.0)	0 (0.0)
Inter-hospital airway					
Endotracheal tube, no. (%), n=193	44 (78.6)	54 (54.0)	18 (62.1)	0 (0.0)	0 (0.0)
Inter-hospital oxygen supplements, n=160					
Nasal cannula, no. (%)	9 (29.0)	21 (22.3)	3 (13.6)	0 (0.0)	3 (33.3)
Venturi mask, no. (%)	1 (3.2)	1 (1.1)	0 (0.0)	0 (0.0)	0 (0.0)
Facial mask, no. (%)	7 (22.6)	2 (2.1)	8 (36.4)	0 (0.0)	1 (11.1)
Bag valve mask, no. (%)	2 (6.5)	13 (13.8)	0 (0.0)	0 (0.0)	0 (0.0)
MV[a], no. (%)	0 (0.0)	41 (43.6)	0 (0.0)	0 (0.0)	0 (0.0)

Others[c], no. (%)	12 (38.7)	5 (5.3)	10 (45.5)	0 (0.0)	0 (0.0)
None of the above, no. (%)	0 (0.0)	11 (11.7)	1 (4.5)	4 (100.0)	5 (55.6)
**Complications**					
Hospital-acquired pneumonia, no. (%)	63 (100.0)	98 (83.8)	30 (100.0)	4 (100.0)	13 (9.4)
Secondary bacterial infections, no. (%)	0 (0.0)	61 (52.1)	0 (0.0)	0 (0.0)	0 (0.0)
Septic shock, no. (%)	53 (84.1)	74 (63.2)	27 (90.0)	3 (75.0)	9 (6.5)
Cardiac injury, no. (%)	14 (22.2)	4 (3.4)	7 (23.3)	1 (25.0)	1 (0.7)
Acute kidney injury, no. (%)	29 (46.0)	51 (43.6)	15 (50.0)	1 (25.0)	5 (3.6)
Liver dysfunction, no. (%)	14 (22.2)	15 (12.8)	10 (33.3)	2 (50.0)	1 (0.7)
Gastrointestinal bleeding, no. (%)	0 (0.0)	0 (0.0)	0 (0.0)	0 (0.0)	1 (0.7)
**Outcome**					
Hospital mortality	34 (54.0)	80 (68.4)	21 (70.0)	2 (50.0)	80 (57.6)

[a] To indicate non-invasive or invasive MV at the referring hospital or during transportation; [b] To indicate family members, relatives, neighbors, layperson, police, or passers-by; [c] To indicate an alternative method for oxygen supplementation, for instance, employing a manual resuscitator bag with an artificial airway.

Abbreviations: **EMS**, Emergency Medical Services; **MV**, mechanical ventilation; **NA**, not available.

S2 Table. Baseline characteristics of patients with acute respiratory distress syndrome, according to hospital survivability

Variables	All cases n=353	Survived n=136	Died n=217	P value[a]
**Demographics**				
Age (year), median (Q1-Q3)	55.0 (39.0-66.0)	44.5 (35.0-61.0)	57.0 (43.0-69.0)	<0.001
Gender (male), no. (%)	240 (68.0)	89 (65.4)	151 (69.6)	0.417
Occupation, n=226				0.262
Farmer, no. (%)	61 (27.0)	28 (34.1)	33 (22.9)	
Government employee, no. (%)	14 (6.2)	5 (6.1)	9 (6.2)	
Commercial, no. (%)	16 (7.1)	6 (7.3)	10 (6.9)	

Office worker, no. (%)	4 (1.8)	3 (3.7)	1 (0.7)	
House wife, no. (%)	13 (5.8)	4 (4.9)	9 (6.2)	
Student, no. (%)	6 (2.7)	2 (2.4)	4 (2.8)	
Retired, no. (%)	54)23.9)	13 (15.9)	41 (28.5)	
Others, no. (%)	58 (25.7)	21 (25.6)	37 (25.7)	
**Habitual behavior**				
Smoking history, n=217				0.383
Never, no. (%)	120 (55.3)	46 (57.5)	74 (54.0)	
Quit, no. (%)	31 (14.3)	8 (10.0)	23 (16.8)	
Current, no. (%)	66 (30.4)	26 (32.5)	40 (29.2)	
**Comorbidities**				
Cerebrovascular disease, no. (%), n=351	7 (2.0)	0 (0.0)	7 (3.2)	0.047
Chronic cardiac failure, no. (%), n=234	10 (4.3)	2 (2.4)	8 (5.3)	0.502
CAD/MI, no. (%), n=222	4 (1.8)	0 (0.0)	4 (2.8)	0.300
Hypertension, no. (%), n=227	67 (29.5)	25 (30.1)	42 (29.2)	0.879
COPD/asthma, no. (%), n=227	13 (5.7)	0 (0.0)	13 (9.0)	0.003
Other CPD, no. (%), n=347	27 (7.8)	12 (9.0)	15 (7.0)	0.517
Tuberculosis, no. (%), n=227	5 (2.2)	1 (1.2)	4 (2.8)	0.655
Active neoplasm, no. (%), n=227	11 (4.8)	2 (2.4)	9 (6.2)	0.336
Chronic renal failure, no. (%), n=231	22 (9.5)	11 (13.3)	11 (7.4)	0.148
Ulcer disease, no. (%), n=350	12 (3.4)	4 (3.0)	8 (3.7)	0.773
Diabetes mellitus, no. (%), n=227	45 (19.8)	16 (19.3)	29 (20.1)	0.875
Immuno compromise, no. (%), n=233	16 (6.9)	7 (8.1)	9 (6.1)	0.557
Hematological disease, no. (%), n=234	17 (7.3)	4 (4.7)	13 (8.7)	0.255
CCI, median (Q1-Q3), n=150	3.0 (1.0-5.0)	2.0 (1.0-4.5)	3.0 (1.0-5.0)	0.310

[a] To indicate comparisons between patients who survived and those who died in the hospital.

Abbreviations: **CAD**, coronary artery disease; **CCI**, Charlson Comorbidity Index; **COPD**, chronic obstructive pulmonary disease; **CPD**, chronic pulmonary disease; **MI**, myocardial infarction; **no.**, number of patients; **Q**, quartile.

S3 Table. Respiratory viral pathogens in patients with acute respiratory distress syndrome, according to hospital survivability

Variables	All cases n=353	Survived n=136	Died n=217	P value[a]
Adenovirus, no. (%), n=223	3 (1.3)	1 (1.2)	2 (1.4)	>0.999
Cytomegalovirus, no. (%), n=221	6 (2.7)	3 (3.8)	3 (2.1)	0.670
Human rhinovirus B, no. (%), n=227	1 (0.4)	1 (1.2)	0 (0.0)	0.366
Parainfluenza virus, no. (%), n=227	3 (1.3)	1 (1.2)	2 (1.4)	>0.999
Influenza virus A (H1N1), no. (%), n=227	29 (12.8)	11 (13.3)	18 (12.5)	0.870
Avian influenza virus A (H5N6), no. (%), n=221	1 (0.5)	0 (0.0)	1 (0.7)	>0.999
Avian influenza virus A (H9N2), no. (%), n=221	1 (0.5)	1 (1.2)	0 (0.0)	0.362
Influenza type B, no. (%), n=227	4 (1.8)	2 (2.4)	2 (1.4)	0.625

[a] To indicate comparisons between patients who survived and those who died in the hospital.

S4 Table. The clinical and laboratory characteristics of patients with acute respiratory distress syndrome upon admission, according to hospital survivability

Variables	All cases n=353	Survived n=136	Died n=217	P value[a]
**Clinical characteristics**				
HR (beats/min), median (Q1-Q3), n=227	123.0 (105.0-138.0)	125.0 (105.0-135.0)	123.0 (106.25-139.5)	0.594
RR (breaths/min), median (Q1-Q3), n=227	27.0 (22.0-31.0)	25.0 (20.0-30.0)	28.0 (23.25-32.0)	0.174
Systolic BP (mmHg), mean (SD), n=227	106.59 (24.75)	111.30 (21.43)	103.87 (26.16)	0.025
Diastolic BP (mmHg), mean (SD), n=227	62.68 (15.22)	66.57 (12.95)	60.44 (16.01)	0.006
Body temperature (^{o}C), mean (SD), n=227	37.83 (1.04)	37.68 (0.9)	37.92 (1.11)	0.167
**Laboratory investigations**				

WBC (x10^9/L), mean (SD), n=352	14.73 (1229)	12.92 (7.71)	15.87 (14.35)	0.131
Lymphocytes (x10^9/L), mean (SD), n=210	7.19 (11.49)	6.59 (7.4)	7.57 (13.42)	0.427
Neutrophils (x10^9/L), mean (SD), n=211	53.73 (37.17)	57.20 (36.56)	51.56 (37.52)	0.453
Hemoglobin (g/L), mean (SD), n=226	115.88 (25.15)	115.06 (22.79)	116.35 (26.49)	0.722
Platelet count (x10^9/L), mean (SD), n=352	178.13 (118.99)	172.3 (97.8)	181.81 (130.67)	0.820
CRP (mg/L), mean (SD), n=84	63.11 (354.96)	21.82 (24.04)	86.05 (442.15)	0.245
Procalcitonin (ng/mL), mean (SD), n=173	30.66 (38.44)	23.55 (32.66)	34.84 (41.03)	0.160
Troponin T (ng/L), mean (SD), n=164	120.69 (267.16)	163.63 (400.39)	97.82 (153.43)	0.919
ProBNP (pg/mL), mean (SD), n=148	1378.22 (4406.96)	830.08 (1794.42)	1675.13 (5298.09)	0.963
Total bilirubin (µmol/L), mean (SD), n=331	23.52 (35.24)	27.82 (46.9)	20.80 (25.03)	0.702
AST (U/L), mean (SD), n=224	179.63 (536.97)	206.76 (781.09)	163.67 (318.17)	0.212
ALT (U/L), mean (SD), n=224	95.01 (206.08)	103.14 (243.60)	90.22 (181.19)	0.256
LDH (U/L), mean (SD), n=4	399.00 (364.75)	709.50 (112.43)	88.50 (28.99)	0.121
Glucose (mmol/L), mean (SD), n=216	10.07 (5.80)	9.39 (4.21)	10.45 (6.52)	0.476
T-chol (mmol/L), mean (SD), n=1	11.23	NA	11.23	NA
LDL-C (mmol/L), mean (SD), n=1	0.73	NA	0.73	NA
Ure (mmol/L), mean (SD), n=226	11.98 (9.49)	9.78 (7.47)	13.26 (10.30)	0.001
Creatinine (µmol/L), mean (SD), n=352	149.78 (147.5)	138.35 (125.18)	156.98 (159.82)	0.143
Albumin (g/L), mean (SD), n=331	26.75 (5.05)	27.73 (4.8)	26.11 (5.12)	0.002
Ferritin (µg/L), mean (SD), n=8	8798.50 (13571.31)	5703.0 (7242.02)	11894.0 (18755.14)	0.386
INR, mean (SD), n=222	1.41 (0.83)	1.29 (0.28)	1.48 (1.03)	0.363
APTT (sec.), mean (SD), n=223	37.82 (16.35)	35.78 (10.78)	39.02 (18.83)	0.369
Fibrinogen (g/L), mean (SD), n=223	5.22 (3.13)	5.54 (3.22)	5.03 (3.08)	0.144
D-dimer (mg/L), mean (SD), n=169	30.28 (236.18)	5.42 (3.58)	46.61 (303.47)	0.683
Interleukin 6 (pg/mL), mean (SD), n=38	751.39 (915.75)	672.72 (1183.03)	815.07 (649.79)	0.181
DIC score, median (Q1-Q3), n=194	3.0 (2.0-4.0)	3.0 (2.0-3.0)	3.0 (2.0-4.0)	0.058

[a] To indicate comparisons between patients who survived and those who died in the hospital.

Abbreviations: **ALT**, alanine transaminase; **APTT**, activated partial thromboplastin time; **AST**, aspartate transaminase; **BP**, blood pressure; **CRP**, C-reactive protein; **DIC**, disseminated intravascular coagulation; **HR**, heart rate; **INR**, international normalized ratio; **LDH**, lactic acid dehydrogenase; **LDL-C**, low-density lipoprotein cholesterol; **NA**, not available; **no.**, number of patients; **ProBNP**, N-terminal pro-brain natriuretic peptide; **Q**, quartile; **RR**, respiration rate; **SD**, standard deviation; **T-chol**, total cholesterol; **WBCs**, white blood cells.

S5 Table. Initial chest imaging findings of patients with acute respiratory distress syndrome, according to hospital survivability

Variables	All cases n=353	Survived n=136	Died n=217	P value[a]
**Chest X-rays**				
Bilateral opacities, no. (%), n=352	350 (99.4)	136 (100.0)	214 (99.1)	0.524
Number of involved quadrants, n=341				0.223
1 quadrant, no. (%)	5 (1.5)	2 (1.5)	3 (1.4)	
2 quadrants, no. (%)	10 (2.9)	4 (3.0)	6 (2.9)	
3 quadrants, no. (%)	88 (25.8)	42 (31.8)	46 (22.0)	
4 quadrants, no. (%)	238 (69.8)	84 (63.6)	154 (73.7)	
**Chest computed tomography scans**				
Honeycombing, no. (%), n=54	25 (46.3)	17 (51.5)	8 (38.1)	0.335
Ground class attenuation, no. (%), n=60	60 (100.0)	36 (100.0)	24 (100.0)	NA
Traction bronchiectasis, no. (%), n=38	12 (31.6)	10 (41.7)	2 (14.3)	0.147
Interlobular septal thickening, no. (%), n=45	33 (73.3)	20 (80.0)	13 (65.0)	0.258
Air space consolidation including atelectasis, no. (%), n=47	42 (89.4)	26 (92.9)	16 (84.2)	0.381

[a] To indicate comparisons between patients who survived and those who died in the hospital.

Abbreviations: **NA**, not available; **no.**, number of patients.

S6 Table. Comparative analysis of prior hospitalization and inter-hospital care in patients with acute respiratory distress syndrome before and during the COVID-19 pandemic

Variables	All cases n=353	Aug 2015 – Dec 2019 n=152	Jan 2020 – Aug 2023 n=201	P value[a]
**Prior hospitalization**				
Prior hospitalization, no. (%)	316 (89.5)	144 (94.7)	172 (85.6)	0.005
Duration of stay (day), mean (SD), n=313	2.73 (3.46)	1.43 (2.02)	3.79 (4.0)	<0.001
MV applied[b], no. (%), n=314	253 (80.6)	139 (97.9)	114 (66.3)	<0.001
Duration of MV (day), mean (SD), n=252	1.45 (1.44)	1.09 (0.29)	1.89 (2.05)	<0.001
**Inter-hospital care**				
The patient was brought in by, n=214				<0.001
EMS, no. (%)	117 (54.7)	26 (100.0)	91 (48.4)	
Hospital ambulances, no. (%)	63 (29.4)	0 (0.0)	63 (33.5)	
Private ambulances, no. (%)	30 (14.0)	0 (0.0)	30 (16.0)	
Public or own/private transport, no. (%)	4 (1.9)	0 (0.0)	4 (2.1)	
Inter-hospital care provider, n=201				<0.001
Bystanders[c], no. (%)	40 (19.9)	0 (0.0)	40 (22.9)	
EMS staff, no. (%)	106 (52.7)	26 (100.0)	80 (45.7)	
Hospital doctors, no. (%)	1 (0.5)	1 (0.6)	0 (0.0)	
Hospital nurses, no. (%)	54 (26.9)	0 (0.0)	54 (30.9)	
Inter-hospital airway				
Endotracheal tube, no. (%), n=193	116 (60.1)	6 (35.3)	110 (62.5)	0.029

[a)] To indicate comparisons between patients who presented to the hospital during the pre-pandemic period of COVID-19 (August 2015 to December 2019) and those who presented to the hospital during the pandemic period of COVID-19 (January 2020 to August 2023). [b)] To indicate non-invasive or invasive MV at the referring hospital or during transportation. [c)] To indicate family members, relatives, neighbors, layperson, police, or passers-by. Abbreviations: **EMS**, Emergency Medical Services; **MV**, mechanical ventilation.

S7 Table. Comparative analysis of demographics and comorbidities in patients with acute respiratory distress syndrome before and during the COVID-19 pandemic

Variables	All cases n=353	Aug 2015 – Dec 2019 n=152	Jan 2020 – Aug 2023 n=201	P value[a]
**Demographics**				
Age (year), median (Q1-Q3)	55.0 (39.0-66.0)	54.0 (39.25-64.75)	55.0 (38.5-66.5)	0.425
Gender (male), no. (%)	240 (68.0)	101 (66.4)	139 (69.2)	0.589
**Comorbidities**				
Cerebrovascular disease, no. (%), n=351	7 (2.0)	5 (3.3)	2 (1.0)	0.142
Chronic cardiac failure, no. (%), n=234	10 (4.3)	9 (27.3)	1 (0.5)	<0.001
CAD/MI, no. (%), n=222	4 (1.8)	0 (0.0)	4 (2.0)	>0.999
Hypertension, no. (%), n=227	67 (29.5)	6 (23.1)	61 (30.3)	0.444
COPD/asthma, no. (%), n=227	13 (5.7)	3 (11.5)	10 (5.0)	0.175
Other CPD, no. (%), n=347	27 (7.8)	23 (15.4)	4 (2.0)	<0.001
Tuberculosis, no. (%), n=227	5 (2.2)	0 (0.0)	5 (2.5)	>0.999
Active neoplasm, no. (%), n=227	11 (4.8)	1 (3.8)	10 (5.0)	>0.999
Chronic renal failure, no. (%), n=231	22 (9.5)	7 (22.6)	15 (7.5)	0.016
Ulcer disease, no. (%), n=350	12 (3.4)	2 (1.3)	10 (5.0)	0.065
Diabetes mellitus, no. (%), n=227	45 (19.8)	8 (30.8)	37 (18.4)	0.137
Immuno compromise, no. (%), n=233	16 (6.9)	8 (25.0)	8 (4.0)	<0.001
Hematological disease, no. (%), n=234	17 (7.3)	9 (26.5)	8 (4.0)	<0.001
CCI Score, median (Q1-Q3), n=150	3.0 (1.0-5.0)	5.0 (3.5-7.0)	2.0 (1.0-4.0)	<0.001

[a)] To indicate comparisons between patients who presented to the hospital during the pre-pandemic period of COVID-19 (August 2015 to December 2019) and those who presented to the hospital during the pandemic period of COVID-19 (January 2020 to August 2023). Abbreviations: **CAD**, coronary artery disease; **CCI**, Charlson Comorbidity Index; **COPD**, chronic obstructive pulmonary disease; **CPD**, chronic pulmonary disease; **MI**, myocardial infarction; **no.**, number of patients; **Q**, quartile.

S8 Table. Comparative analysis of respiratory viral pathogens in patients with acute respiratory distress syndrome before and during the COVID-19 pandemic

Variables	All cases n=353	Aug 2015 – Dec 2019 n=152	Jan 2020 – Aug 2023 n=201	P value[a]
Adenovirus, no. (%), n=223	3 (1.3)	0 (0.0)	3 (1.5)	>0.999
Cytomegalovirus, no. (%), n=221	6 (2.7)	0 (0.0)	6 (3.1)	>0.999
Human rhinovirus B, no. (%), n=227	1 (0.4)	0 (0.0)	1 (0.5)	>0.999
Parainfluenza virus, no. (%), n=227	3 (1.3)	0 (0.0)	3 (1.5)	>0.999
Influenza virus A (H1N1), no. (%), n=227	29 (12.8)	20 (76.9)	9 (4.5)	<0.001
Avian influenza virus A (H5N6), no. (%), n=221	1 (0.5)	0 (0.0)	1 (0.5)	>0.999
Avian influenza virus A (H9N2), no. (%), n=221	1 (0.5)	0 (0.0)	1 (0.5)	>0.999
Influenza type B, no. (%), n=227	4 (1.8)	1 (3.8)	3 (1.5)	0.387

[a] To indicate comparisons between patients who presented to the hospital during the pre-pandemic period of COVID-19 (August 2015 to December 2019) and those who presented to the hospital during the pandemic period of COVID-19 (January 2020 to August 2023).

S9 Table. Comparative analysis of clinical and laboratory characteristics in patients with acute respiratory distress syndrome upon admission before and during the COVID-19 pandemic

Variables	All cases n=353	Aug 2015 – Dec 2019 n=152	Jan 2020 – Aug 2023 n=201	P value[a]
**Etiology**				
Etiology of ARDS, no. (%), n=226				
Pneumonia	205 (90.7)	26 (100.0)	179 (89.5)	0.143
Aspiration of gastric contents	5 (2.2)	0 (0.0)	5 (2.5)	>0.999
Pulmonary contusion n=225	2 (0.9)	0 (0.0)	2 (1.0)	>0.999

Inhalation injury	3 (1.3)	0 (0.0)	3 (1.5)	>0.999
Pulmonary vasculitis	1 (0.4)	0 (0.0)	1 (0.5)	>0.999
Drowning n=227	9 (4.0)	0 (0.0)	9 (4.5)	0.603
**Clinical characteristics**				
HR (beats/min), median (Q1-Q3), n=227	123.0 (105.0 -138.0)	115.0 (100.0-130.0)	125.0 (110.0-138.0)	0.191
RR (breaths/min), median (Q1-Q3), n=227	27.0 (22.0-31.0)	33.5 (27.25-35.0)	26.0 (21.0-30.0)	<0.001
Systolic BP (mmHg), mean (SD), n=227	106.59 (24.75)	109.81 (20.12)	106.17 (25.3)	0.482
Diastolic BP (mmHg), mean (SD), n=227	62.68 (15.22)	65.77 (11.38)	62.28 (15.63)	0.273
Body temperature (^{o}C), mean (SD), n=227	37.83 (1.04)	37.89 (0.93)	37.82 (1.06)	0.739
**Laboratory investigations**				
WBCs (x10^9/L), mean (SD), n=352	14.73 (12.29)	14.24 (12.57)	15.10 (12.09)	0.155
Hemoglobin (g/L), mean (SD), n=226	115.88 (25.15)	111.69 (21.32)	116.42 (25.6)	0.368
Platelet count (x10^9/L), mean (SD), n=352	178.13 (118.99)	180.89 (116.08)	176.04 (121.40)	0.598
CRP (mg/L), mean (SD), n=84	63.11 (354.96)	118.20 (565.59)	27.46 (33.48)	0.608
Total bilirubin (µmol/L), mean (SD), n=331	23.52 (35.24)	23.32 (40.52)	23.66 (30.96)	0.724
Glucose (mmol/L), mean (SD), n=216	10.07 (5.80)	10.77 (4.89)	9.97 (5.92)	0.509
Ure (mmol/L), mean (SD), n=226	11.98 (9.49)	12.31 (11.20)	11.94 (9.28)	0.613
Creatinine (µmol/L), mean (SD), n=352	149.78 (147.5)	149.91 (171.43)	149.69 (126.77)	0.566

[a)] To indicate comparisons between patients who presented to the hospital during the pre-pandemic period of COVID-19 (August 2015 to December 2019) and those who presented to the hospital during the pandemic period of COVID-19 (January 2020 to August 2023). Abbreviations: **BP**, blood pressure; **CRP**, C-reactive protein; **HR**, heart rate; **no.**, number of patients; **Q**, quartile; **RR**, respiration rate; **SD**, standard deviation; **WBCs**, white blood cells.

S10 Table. Comparative analysis of arterial blood gas parameters, chest x-ray findings, and severity of illness upon admission in patients with acute respiratory distress syndrome pre- and during the COVID-19 pandemic

Variables	All cases<br>n=353	Aug 2015 – Dec 2019<br>n=152	Jan 2020 – Aug 2023<br>n=201	P value[a]
**Arterial blood gas**				
pH, mean (SD), n=350	7.32 (0.16)	7.3 (0.13)	7.3 (0.17)	0.003
PaO_2 (mmHg), mean (SD), n=349	81.29 (35.25)	81.88 (35.92)	80.84 (34.82)	0.785
$PaCO_2$ (mmHg), mean (SD), n=350	44.17 (16.1)	42.92 (13.88)	45.11 (17.57)	0.462
SpO_2 (%), mean (SD), n=348	92.24 (49.03)	95.22 (74.6)	90.02 (6.42)	0.328
PaO_2/FiO_2 ratio (mmHg), mean (SD), n=350	110.04 (57.72)	110.79 (58.12)	109.48 (57.55)	0.834
**Initial chest X-ray findings**				
Bilateral opacities, no. (%), n=352	350 (99.4)	149 (98.7)	201 (100.0)	0.183
Number of involved quadrants, n=341				<0.001
1 quadrant, no. (%)	5 (1.5)	5 (3.6)	0 (0.0)	
2 quadrants, no. (%)	10 (2.9)	4 (2.9)	6 (3.0)	
3 quadrants, no. (%)	88 (25.8)	17 (12.1)	71 (35.3)	
4 quadrants, no. (%)	238 (69.8)	114 (81.4)	124 (61.7)	
**Severity of illness**				
Berlin criteria, no. (%), n=350				0.410
$200 \text{ mmHg} < PaO_2/FiO_2 \leq 300 \text{ mmHg}$	33 (9.4)	14 (9.3)	19 (9.5)	
$100 \text{ mmHg} < PaO_2/FiO_2 \leq 200 \text{ mmHg}$	121 (34.6)	58 (38.4)	63 (31.7)	
$PaO_2/FiO_2 \leq 100 \text{ mmHg}$	196 (56.0)	79 (52.3)	117 (58.8)	
SOFA, median (Q1-Q3), n=335	10.0 (7.0-12.0)	8.5 (6.0-11.0)	10.0 (7.0-12.0)	0.004

[a)] To indicate comparisons between patients who presented to the hospital during the pre-pandemic period of COVID-19 (August 2015 to December 2019) and those who presented to the hospital during the pandemic period of COVID-19 (January 2020 to August 2023). Abbreviations: **$PaCO_2$**, partial pressure of carbon dioxide in the arterial blood; **PaO_2**, partial pressure of oxygen in the arterial blood; **pH**, the acidity of the blood; **no.**, number of patients; **SD**, standard deviation; **SOFA Score**, Sequential Organ

Failure Assessment Score; **SpO_2**, saturation of oxygen in the peripheral blood.

S11 Table. Comparative analysis of management strategies for patients with acute respiratory distress syndrome before and during the COVID-19 pandemic

Variables	All cases n=353	Aug 2015 – Dec 2019 n=152	Jan 2020 – Aug 2023 n=201	P value[a]
**Respiratory support**				
The first day respiratory support, n=329				0.005
Oxygen only, no. (%)	7 (2.1)	0 (0.0)	7 (3.5)	
Non-invasive MV, no. (%)	7 (2.1)	0 (0.0)	7 (3.5)	
Invasive MV, no. (%)	315 (95.7)	129 (100.0)	186 (93.0)	
**Adjunctive therapies during ICU stay**				
Prone positioning, no. (%), n=345	85 (24.6)	32 (21.3)	53 (27.2)	0.212
Recruitment maneuvers, no. (%), n=348	60 (17.2)	29 (19.3)	31 (15.7)	0.369
ECMO, no. (%), n=263	22 (8.4)	9 (6.8)	13 (10.0)	0.344
Antiviral drugs, no. (%), n=335	70 (20.9)	48 (34.0)	22 (11.3)	<0.001
Antibiotics, no. (%), n=335	328 (97.9)	141 (100.0)	187 (96.4)	0.023
Corticosteroids, no. (%), n=342	82 (24.0)	12 (8.0)	70 (36.5)	<0.001
Continuous sedation, no. (%), n=349	333 (95.4)	145 (96.7)	188 (94.5)	0.332
NMBAs, no. (%), n=345	247 (71.6)	95 (63.8)	152 (77.6)	0.005
RRT, no. (%), n=138	138 (100.0)	8 (100.0)	130 (100.0)	NA
ECAT, no. (%), n=199	73 (36.7)	1 (5.9)	72 (39.6)	0.006
Tracheostomy, no. (%), n=263	16 (6.1)	6 (4.5)	10 (7.7)	0.281
Inhaled vasodilators, no. (%), n=227	1 (0.4)	0 (0.0)	1 (0.5)	>0.999
Neutrophil elastase therapy, no. (%), n=226	1 (0.4)	0 (0.0)	1 (0.5)	>0.999

[a)] To indicate comparisons between patients who presented to the hospital during the pre-pandemic period of COVID-19 (August 2015 to December 2019) and those who presented to the hospital during the pandemic period of COVID-19 (January 2020 to August 2023). Abbreviations: **ECAT**, extracorporeal cytokine adsorption therapy; **ECMO**, extracorporeal membrane oxygenation; **MV**, mechanical ventilation; **NA**, not available; **NMBAs**, neuromuscular blocking agents; **no.**, number of patients; **RRT**, renal replacement therapy; **SD**, standard deviation.

S12 Table. Comparative analysis of clinical time course and complications in patients with acute respiratory distress syndrome pre- and during the COVID-19 pandemic

Variables	All cases n=353	Aug 2015 – Dec 2019 n=152	Jan 2020 – Aug 2023 n=201	P value[a]
**Outcome**				
Died in the hospital	217 (61.5)	91 (59.9)	126 (62.7)	0.590
**Clinical time-course**				
LOS (day), mean (SD), n=348	10.19 (11.54)	10.55 (10.8)	9.92 (12.09)	0.345
**Complications**				
HAP, no. (%)	208 (58.9)	17 (11.2)	191 (95.0)	<0.001
Secondary bacterial infections, no. (%)	61 (17.3)	10 (6.6)	51 (25.4)	<0.001
Septic shock, no. (%)	166 (47.0)	10 (6.6)	156 (77.6)	<0.001
Cardiac injury, no. (%)	27 (7.6)	0 (0.0)	27 (13.4)	<0.001
Acute kidney injury, no. (%)	101 (28.6)	7 (4.6)	94 (46.8)	<0.001
Liver dysfunction, no. (%)	42 (11.9)	3 (2.0)	39 (19.4)	<0.001
Gastrointestinal bleeding, no. (%)	1 (0.3)	0 (0.0)	1 (0.5)	>0.999

[a)] To indicate comparisons between patients who presented to the hospital during the pre-pandemic period of COVID-19 (August 2015 to December 2019) and those who presented to the hospital during the pandemic period of COVID-19 (January 2020 to August 2023). Abbreviations: **HAP**, hospital-acquired pneumonia; **ICU**, intensive care unit; **LOS**, hospital lengths of stay; **no.**, number of patients; **SD**, standard deviation.

S13 Table. Overall diagnostic performance of the Charlson Comorbidity Index, PaO_2/FiO_2 ratio, and Sequential Organ Failure Assessment Score upon admission for predicting hospital mortality in patients with acute respiratory distress syndrome

Variables	AUROC (95% CI)	p-value	Cut-off value	Sensitivity (%)	Specificity (%)
CCI	0.549 (0.451-0.647)	0.321	7.5	4.10	100
PaO_2/FiO_2 ratio	0.592 (0.528- 0.656)	0.004	121.1 mmHg	75.7	46.3
SOFA Score	0.651 (0.591-0.710)	<0.001	9.50	60.7	63.6

Abbreviations: **AUROC**, Area Under the Receiver Operating Characteristic curve; **CCI**, Charlson Comorbidity Index; **CI**, confidence interval; **PaO_2/FiO_2 ratio**, the ratio of arterial oxygen partial pressure to fractional-inspired oxygen; **SOFA Score**, Sequential Organ Failure Assessment Score.

S14 Table. Factors associated with hospital mortality in patients with acute respiratory distress syndrome

Factors	Univariable logistic regression analyses[a]				Multivariable logistic regression analysis[b]			
	OR	95% CI for OR		p-value	AOR	95% CI for AOR		p-value
		Lower	Upper			Lower	Upper	
**Prior hospitalization**								
MV[c]	0.960	0.542	1.701	0.889	NA	NA	NA	NA
**Inter-hospital care**								
The patient was brought in by								
EMS	Reference			0.215	NA			NA
Hospital ambulances	0.542	0.289	1.018	0.057	NA	NA	NA	NA
Private ambulances	1.079	0.451	2.583	0.864	NA	NA	NA	NA
Inter-hospital care provider								
Bystanders[d]	Reference			0.505	NA			NA
EMS staff	1.635	0.773	3.460	0.199	NA	NA	NA	NA
Hospital nurses	1.075	0.469	2.464	0.864	NA	NA	NA	NA
Inter-hospital airway								

Endotracheal tube	0.619	0.337	1.136	0.122	0.057	0.004	0.887	0.041
Inter-hospital oxygen								
Nasal cannula	Reference			0.429	NA			NA
Facial mask	0.867	0.241	3.110	0.826	NA	NA	NA	NA
Bag valve mask	0.292	0.082	1.033	0.056	NA	NA	NA	NA
Mechanical ventilator[c]	0.386	0.146	1.021	0.055	NA	NA	NA	NA
Others[e]	0.576	0.191	1.679	0.305	NA	NA	NA	NA
None of the above	0.542	0.170	1.727	0.300	NA	NA	NA	NA
**Demographics**								
Age (year)	1.027	1.013	1.040	<0.001	NA	NA	NA	NA
Gender (male)	0.828	0.524	1.307	0.417	NA	NA	NA	NA
**Documented Comorbidities**								
Chronic cardiac failure	2.310	0.479	11.138	0.297	NA	NA	NA	NA
Active neoplasm	2.700	0.569	12.807	0.221	NA	NA	NA	NA
Chronic renal failure	0.526	0.217	1.271	0.153	NA	NA	NA	NA
Hematological disease	1.936	0.611	6.137	0.262	NA	NA	NA	NA
CCI Score	1.086	0.914	1.290	0.347	0.576	0.273	1.213	0.147
**Severity of illness**								
PaO_2/FiO_2 ratio	0.993	0.989	0.996	<0.001	NA	NA	NA	NA
SOFA Score $\geq$ 9.5[f]	2.692	1.709	4.242	<0.001	14.819	1.410	155.760	0.025
**Adjunctive therapies**								
Recruitment maneuver	1.310	0.729	2.355	0.366	NA	NA	NA	NA
Corticosteroid	0.884	0.532	1.468	0.633	NA	NA	NA	NA
ECAT	0.924	0.510	1.676	0.795	18.259	1.038	321.089	0.047
Tracheostomy	0.198	0.062	0.632	0.006	NA	NA	NA	NA
**Complications**								
HAP	1.058	0.684	1.635	0.801	NA	NA	NA	NA

Secondary bacterial infections	2.170	1.159	4.064	0.016	NA	NA	NA	NA
Septic shock	2.077	1.338	3.226	0.001	16.951	0.864	332.669	0.062
Constant					0.858			0.912

[a] Each variable of the inter-hospital care, demographics, documented comorbidities, clinical and laboratory features, gas exchange, chest X-ray findings, severity of illness, oxygen supplement, MV, adjunctive therapies, and complications was analyzed in the univariable logistic regression model and was considered in the multivariable logistic regression model if the P-value was <0.05 in univariable logistic regression analysis, as well as clinically crucial factors.

[b] All selected variables were included in the multivariable logistic regression model with the stepwise backward elimination method. Variables, then, were deleted stepwise from the full model until all remaining variables were independently associated with hospital mortality.

[c] To indicate non-invasive or invasive MV at the referring hospital or during transportation.

[d] To indicate family members, relatives, neighbors, layperson, police, or passers-by.

[e] To indicate an alternative method for oxygen supplementation, for instance, employing a manual resuscitator bag with an artificial airway.

[f] To indicate the best cut-off value determined by analyzing the receiver operator characteristic curve of SOFA Score for predicting hospital mortality.

Abbreviations: **AOR**, adjusted odds ratio; **CCI**, Charlson Comorbidity Index; **CI**, confidence interval; **ECAT**, extracorporeal cytokine adsorption therapy; **EMS**, emergency medical services; **HAP**, hospital-acquired pneumonia; **MV**, mechanical ventilation; **NA**, not available; **OR**, odds ratio; **PaO_2/FiO_2**, the ratio of arterial oxygen partial pressure to fractional-inspired oxygen; **SOFA Score**, Sequential Organ Failure Assessment Score.

S15 Table. Management strategies for patients with acute respiratory distress syndrome, according to the severity of illness upon admission

Variables	All cases (n=335)	SOFA<9.5 (n=163)	SOFA≥9.5 (n=172)	P value[a]
**Respiratory support**				
The first day respiratory support, n=318				<0.001
Oxygen only, no. (%)	7(2.2)	7(4.5)	0	
Non-invasive MV, no. (%)	7(2.2)	7(4.5)	0	
Invasive MV, no. (%)	304 (95.6)	140 (90.9)	164 (100)	
**Adjunctive therapies during ICU stay**				
Prone positioning, no. (%), n=330	82 (24.8)	34 (21.1)	48 (28.4)	0.126
Recruitment maneuvers, no. (%), n=333	59 (17.7)	27 (16.6)	32 (18.8)	0.589
ECMO, no. (%), n=249	22 (8.8)	11 (8.9)	11 (8.8)	0.984

Antiviral drugs, no. (%), n=321	69 (21.5)	41 (26.3)	28 (17.0)	0.042
Antibiotics, no. (%), n=321	314 (97.8)	154 (98.7)	160 (97.0)	0.449
Corticosteroids, no. (%), n=327	81 (24.8)	35 (21.7)	46 (27.7)	0.211
Continuous sedation, no. (%), n=334	320 (95.8)	157 (96.3)	163 (95.3)	0.649
NMBAs, no. (%), n=331	240 (72.5)	115 (71.4)	125 (73.5)	0.669
RRT, no. (%), n=138	138 (100.0)	52 (100.0)	86 (100.0)	NA
ECAT, no. (%), n=198	73 (36.9)	25 (29.8)	48 (42.1)	0.075
Tracheostomy, no. (%), n=249	16 (6.4)	7 (5.6)	9 (7.2)	0.617
Inhaled vasodilators, no. (%), n=224	1 (0.4)	0 (0.0)	1 (0.8)	>0.999
Neutrophil elastase therapy, no. (%), n=223	1 (0.4)	0 (0.0)	1 (0.8)	>0.999
**Outcome**				
Death	206 (61.5)	81 (49.7)	125 (72.7)	<0.001

[a)] To indicate comparisons between patients who presented with a SOFA score <9.5 and those who presented with a SOFA score ≥9.5 upon admission. Abbreviations: **ECAT**, extracorporeal cytokine adsorption therapy; **ECMO**, extracorporeal membrane oxygenation; **MV**, mechanical ventilation; **NA**, not available; **NMBAs**, neuromuscular blocking agents; **no.**, number of patients; **RRT**, renal replacement therapy; **SD**, standard deviation.

S16 Table. Management strategies for patients with acute respiratory distress syndrome, according to the severity of hypoxemia upon admission

Variables	All cases (n=350)	PaO_2/FiO_2<121.1 mmHg (n=235)	PaO_2/FiO_2≥121.1 mmHg (n=115)	P value[a]
**Respiratory support**				
The first day respiratory support, n=328				0.608
Oxygen only, no. (%)	7 (2.1)	4 (1.8)	3 (2.8)	
Non-invasive MV, no. (%)	7 (2.1)	4 (1.8)	3 (2.8)	
Invasive MV, no. (%)	304 (95.7)	214 (96.4)	100 (94.3)	
None of the above, no. (%)				
**Adjunctive therapies during ICU stay**				

Prone positioning, no. (%), n=343	84 (24.5)	64 (27.7)	20 (17.9)	0.047
Recruitment maneuvers, no. (%), n=346	59 (17.1)	39 (16.7)	20 (17.7)	0.824
ECMO, no. (%), n=262	22 (8.4)	19 (11.0)	3 (3.4)	0.035
Antiviral drugs, no. (%), n=333	70 (21.0)	46 (20.4)	24 (22.4)	0.664
Antibiotics, no. (%), n=333	326 (97.9)	219 (96.9)	107 (100)	0.101
Corticosteroids, no. (%), n=340	81 (23.8)	50 (21.9)	31 (27.7)	0.242
Continuous sedation, no. (%), n=347	332 (95.7)	225 (96.6)	107 (93.9)	0.267
NMBAs, no. (%), n=343	246 (71.7)	177 (77.3)	69 (60.5)	0.001
RRT, no. (%), n=138	138 (100.0)	97 (100.0)	41 (100.0)	NA
ECAT, no. (%), n=197	73 (37.1)	50 (36.8)	23 (37.7)	0.899
Tracheostomy, no. (%), n=262	16 (1.1)	11 (6.4)	5 (5.6)	0.813
Inhaled vasodilators, no. (%), n=225	1 (0.4)	1 (0.6)	0 (0.0)	>0.999
Neutrophil elastase therapy, no. (%), n=224	1 (0.4)	1 (0.6)	0 (0.0)	>0.999
**Outcome**				
Died in the hospital	214 (61.1)	162 (68.9)	52 (45.2)	<0.001

[a)] To indicate comparisons between patients who presented with a PaO_2/FiO_2 ratio <121.1 mmHg and those who presented with a PaO_2/FiO_2 ratio ≥121.1 mmHg upon admission. Abbreviations: **ECAT**, extracorporeal cytokine adsorption therapy; **ECMO**, extracorporeal membrane oxygenation; **MV**, mechanical ventilation; **NA**, not available; **NMBAs**, neuromuscular blocking agents; **no.**, number of patients; **RRT**, renal replacement therapy; **SD**, standard deviation.

S17 Table. Breakdown of missing data

Characteristics	All cases (n=353)
**Prior hospitalization**	
Prior hospitalization	0
Duration of stay (day)	40
MV applied[a]	39
Duration of MV (day)	101
**Inter-hospital care**	
The patient was brought in by	139
EMS	
Hospital ambulances	
Private ambulances	
Public or own/private transport	
Inter-hospital care provider	152
Bystanders[b]	
EMS staff	
Hospital doctors	
Hospital nurses	
Inter-hospital airway	
Endotracheal tube	160
Inter-hospital oxygen supplements	193
Nasal cannula	
Venturi mask	
Facial mask	
Bag valve mask	
MV[a]	
Others[c]	
None of the above	
**Demographics**	
Age (year)	0
Gender (male)	0
Occupation	127
Farmer	
Government employee	
Commercial	
Office worker	
House wife	
Student	
Retired	
Others	

**Habitual behavior**	
Smoking history	136
Never	
Quit	
Current	
**Comorbidities**	
Cerebrovascular disease	2
Chronic cardiac failure	119
CAD/MI	131
Hypertension	126
COPD/asthma	126
Other CPD	6
Tuberculosis	126
Active neoplasm	126
Chronic renal failure	122
Ulcer disease	3
Diabetes mellitus	126
Immuno compromise	120
Hematological disease	119
CCI	203
**Etiology of ARDS**	
Pneumonia	127
Aspiration of gastric contents	127
Pulmonary contusion	128
Inhalation injury	127
Pulmonary vasculitis	127
Drowning	126
**Respiratory viral pathogens**	
Adenovirus	130
Cytomegalovirus	132
Human rhinovirus B	126
Parainfluenza virus	126
Influenza virus A (H1N1)pdm09	126
Avian influenza virus A (H5N6)	132
Avian influenza virus A (H9N2)	132
Influenza type B	126
**Initial chest imaging findings**	
Chest X-rays	
Bilateral opacities	1
Number of involved quadrants	12
1 quadrant	
2 quadrants	

3 quadrants	
4 quadrants	
Chest computed tomography scans	
Honeycombing	299
Ground class attenuation	293
Traction bronchiectasis	315
Interlobular septal thickening	308
Air space consolidation including atelectasis	306
**Clinical characteristics**	
HR (beats/min)	126
RR (breaths/min)	126
Systolic BP (mmHg)	126
Diastolic BP (mmHg)	126
Body temperature (^oC)	126
**Laboratory investigations**	353
WBC ($x10^9/L$)	1
Hemoglobin (g/L)	127
Platelet count ($x10^9/L$)	1
CRP (mg/L)	269
Procalcitonin (ng/mL)	180
Troponin T (ng/L)	189
Total bilirubin (μmol/L)	22
AST (U/L)	129
ALT (U/L)	129
Glucose (mmol/L)	137
Ure (mmol/L)	127
Creatinine (μmol/L)	1
INR	131
Interleukin 6 (pg/mL)	315
**Gas exchange**	353
pH	3
PaO_2 (mmHg)	4
$PaCO_2$ (mmHg)	3
FiO_2 (%)	7
PaO_2/FiO_2 ratio	3
SpO_2 (%)	5
**Severity of illness**	
Berlin criteria	3
200 mmHg < $PaO_2/FiO_2 \leq$ 300 mmHg	
100 mmHg < $PaO_2/FiO_2 \leq$ 200 mmHg	
$PaO_2/FiO_2 \leq$ 100 mmHg	
SOFA Score	18

**Respiratory support**	
The first day respiratory support	24
Oxygen only	
Non-invasive MV	
Invasive MV	
The third day respiratory support	99
Oxygen only	
Non-invasive MV	
Invasive MV	
**Adjunctive therapies**	
Prone positioning	8
Recruitment maneuvers	5
ECMO	90
Antiviral drugs	18
Antibiotics	18
Corticosteroids	11
Continuous sedation	4
NMBAs	8
RRT	215
ECAT	154
Tracheostomy	90
Inhaled vasodilators	126
Neutrophil elastase therapy	127
**Complications**	
HAP	0
Secondary bacterial infections	0
Septic shock	0
Cardiac injury	0
Acute kidney injury	0
Liver dysfunction	0
Gastrointestinal bleeding	0

[a] To indicate non-invasive or invasive MV at the referring hospital or during transportation; [b] To indicate family members, relatives, neighbors, layperson, police, or passers-by; [c] To indicate an alternative method for oxygen supplementation, for instance, employing a manual resuscitator bag with an artificial airway. Abbreviations: **ALT**, alanine transaminase; **ARDS**, acute respiratory distress syndrome; **AST**, aspartate transaminase; **BP**, blood pressure; **CAD**, coronary artery disease; **CCI**, Charlson Comorbidity Index; **COPD**, chronic obstructive pulmonary disease; **CPD**, chronic pulmonary disease; **CRP**, C-reactive protein; **ECAT**, extracorporeal cytokine adsorption therapy; **ECMO**, extracorporeal membrane oxygenation; **EMS**, Emergency Medical Services; **FiO_2**, the fraction of inspired oxygen; **HAP**, hospital-acquired pneumonia; **HR**, heart rate; **INR**, international normalized ratio; **MI**, myocardial infarction; **MV**, mechanical ventilation; **NMBAs**, neuromuscular blocking agents; **$PaCO_2$**, partial pressure of carbon dioxide in the arterial blood; **PaO_2**, partial pressure of oxygen in the arterial blood; **PaO_2/FiO_2 ratio**, the ratio of arterial oxygen partial pressure to fractional inspired oxygen; **pH**, the acidity of the blood; **RR**, respiration rate; **RRT**, renal replacement therapy; **SOFA Score**, Sequential Organ Failure Assessment Score; **SpO_2**, saturation of oxygen in the peripheral blood; **WBCs**, white blood cells.
