# Supplementary material for: Factors related to mortality in patients with acute respiratory distress syndrome (ARDS) in a lower middle-income country: A retrospective observational study
Source: PLoS One. 2025 Nov 18;20(11):e0337071. doi: 10.1371/journal.pone.0337071 (PMC12626306; doi:10.1371/journal.pone.0337071)
Supplement: Attachment — Submitted filename: Response to Reviewers.pdf [file pone.0337071.s003.pdf]

Dr. Aliasghar Karimi
Academic Editor
PLOS ONE

December 16, 2024

Dear Dr. Aliasghar Karimi,

On behalf of all the authors, we are resubmitting our revised manuscript titled *"Factors related to mortality in patients with acute respiratory distress syndrome (ARDS) in a lower middle-income country: a retrospective observational study"* (PONE-D-24-35750).

We sincerely appreciate the thoughtful comments and suggestions provided by the Editors and Reviewers. We have carefully addressed all feedback and revised our manuscript accordingly. These insights have significantly improved the quality of our work, and we hope the Editor finds our revised manuscript suitable for publication in PLOS ONE.

We confirm that this research is original, has not been published elsewhere, and is not currently under consideration by any other journal. All authors have read and approved the manuscript, have agreed on the order of authorship, and hold the necessary permissions and rights for the data presented.

Below, we have included our point-by-point responses to the comments from the Editor and the Reviewers. Thank you for your kind consideration of this submission.

Sincerely,

Chinh Quoc Luong, MD, PhD
Center for Emergency Medicine,
Bach Mai Hospital,
No. 78, Giai Phong, Dong Da district,
Hanoi 100000, Vietnam
Email: luongquocchinh@gmail.com

Son Ngoc Do, MD, PhD
Center for Critical Care Medicine,
Bach Mai Hospital,

No. 78, Giai Phong Road, Dong Da District,
Hanoi, 100000, Vietnam.
Email: sonngocdo@gmail.com

*****

We thank the Editors and the Reviewers for the valuable comments and suggestions that have helped us improve the contents of this paper. In what follows, we have used the **boldface** to indicate the Editors' and the Reviewers' comments and the standard font face for our responses. We also highlighted in yellow the modifications that we performed on the manuscript.

RESPONSE TO EDITOR COMMENTS

PONE-D-24-35750

Factors related to mortality in patients with acute respiratory distress syndrome (ARDS) in a lower middle-income country: a retrospective observational study

PLOS ONE

Dear Dr. Do,

Thank you for submitting your manuscript to PLOS ONE. After careful consideration, we feel that it has merit but does not fully meet PLOS ONE's publication criteria as it currently stands. Therefore, we invite you to submit a revised version of the manuscript that addresses the points raised during the review process.

Our answer:

We appreciate your positive feedback. We have thoughtfully addressed the Editors' and Reviewers' comments and suggestions and revised our manuscript accordingly.

Please submit your revised manuscript by Dec 29 2024 11:59PM. If you will need more time than this to complete your revisions, please reply to this message or contact the journal office at plosone@plos.org. When you're ready to submit your revision, log on

to https://www.editorialmanager.com/pone/ and select the 'Submissions Needing Revision' folder to locate your manuscript file.

Our answer:

Thank you for your comment. We have submitted the revised manuscript before the deadline.

Please include the following items when submitting your revised manuscript:

- **A rebuttal letter that responds to each point raised by the academic editor and reviewer(s). You should upload this letter as a separate file labeled 'Response to Reviewers'.**
- **A marked-up copy of your manuscript that highlights changes made to the original version. You should upload this as a separate file labeled 'Revised Manuscript with Track Changes'.**
- **An unmarked version of your revised paper without tracked changes. You should upload this as a separate file labeled 'Manuscript'.**

Our answer:

Thank you for your comment. When submitting our revised manuscript, we included a rebuttal letter, a marked-up copy of the manuscript, and an unmarked version of the revised manuscript.

If you would like to make changes to your financial disclosure, please include your updated statement in your cover letter. Guidelines for resubmitting your figure files are available below the reviewer comments at the end of this letter.

Our answer:

Thank you for your comment. We confirm that there are no changes to our financial disclosure.

If applicable, we recommend that you deposit your laboratory protocols in protocols.io to enhance the reproducibility of your results. Protocols.io assigns your protocol its own identifier (DOI) so that it can be cited independently in the future.

For instructions see: https://journals.plos.org/plosone/s/submission-guidelines#loc-laboratory-protocols.

Additionally, PLOS ONE offers an option for publishing peer-reviewed Lab Protocol articles, which describe protocols hosted on protocols.io. Read more information on sharing protocols at: https://plos.org/protocols?utm_medium=editorial-email&utm_source=authorletters&utm_campaign=protocols.

Our answer:

Thank you for your comment. While laboratory protocol does not apply to our study, the study protocol does. We have included our study protocol in the Methods section.

We look forward to receiving your revised manuscript.

Kind regards,

Aliasghar Karimi
Academic Editor
PLOS ONE

Our answer:

Thank you to the Editor and Reviewers for their support and for taking the time to provide their high-quality reviews.

Journal Requirements:

1. When submitting your revision, we need you to address these additional requirements.

Please ensure that your manuscript meets PLOS ONE's style requirements, including those for file naming. The PLOS ONE style templates can be found at

https://journals.plos.org/plosone/s/file?id=wjVg/PLOSOne_formatting_sample_main_body.pdf and

https://journals.plos.org/plosone/s/file?id=ba62/PLOSOne_formatting_sample_title_authors_affiliations.pdf

Our answer:
Thank you for your comment. We confirm that our manuscript adheres to PLOS ONE's style requirements, including the guidelines for file naming.

2. We noticed you have some minor occurrence of overlapping text with the following previous publication(s), which needs to be addressed:
https://bmjopen.bmj.com/content/13/3/e064870.full

In your revision ensure you cite all your sources (including your own works), and quote or rephrase any duplicated text outside the methods section. Further consideration is dependent on these concerns being addressed.

Our answer:

Thank you for your comment. We have addressed minor occurrences of overlapping text with a previous publication. Additionally, we confirm that all sources, including our works, have been appropriately cited, and any duplicated text outside the Methods section has been quoted or rephrased. We hope this meets your expectations.

3. We noted in your submission details that a portion of your manuscript may have been presented or published elsewhere. This retrospective observational study is the major update of our previously published paper,[1] which collected data on all ARDS patients admitted to the Bach Mai Hospital (BMH) in Hanoi, Vietnam, between August 2015 and August 2017 to elucidate the clinical epidemiology and disease prognosis in ARDS patients in Vietnam. To further investigate the mortality rate and associated

factors from ARDS, especially those related to patient transportation, we continued to collect retrospective data on these patients admitted to the BMH between September 2017 and August 2023, following approval from the BMH Scientific and Ethics Committees (08/11/2023). Subsequently, we merged the data sets from the two stages of data collection at this hospital.

[1] Chinh LQ, Manabe T, Son DN, Chi NV, Fujikura Y, Binh NG, Co DX, Tuan DQ, Ton MD, Dai KQ, Thach PT, Nagase H, Kudo K, Nguyen DA. Clinical epidemiology and mortality on patients with acute respiratory distress syndrome (ARDS) in Vietnam. PLoS One. 2019 Aug 15;14(8):e0221114. doi: 10.1371/journal.pone.0221114. PMID: 31415662; PMCID: PMC6695190. Please clarify whether this [conference proceeding or publication] was peer-reviewed and formally published. If this work was previously peer-reviewed and published, in the cover letter please provide the reason that this work does not constitute dual publication and should be included in the current manuscript.

Our answer:

Thank you for your comment. In the cover letter, we have explained why this work does not constitute dual publication and should be included in the current manuscript. The explanation is also provided below:

This retrospective observational study updates our previous research on ARDS patients at Bach Mai Hospital (BMH) in Hanoi, Vietnam [1]. Initially, data were collected from August 2015 to August 2017. To further investigate ARDS mortality and factors related to patient transportation, we extended data collection to include patients admitted between September 2017 and August 2023, with approval from the BMH Scientific and Ethics Committees (08/11/2023). We merged these datasets and incorporated additional data not included in the previous studies. This submission also presents new, unpublished data. Therefore, this work does not constitute dual publication and should be included in the current manuscript.

[1] Chinh LQ, Manabe T, Son DN, Chi NV, Fujikura Y, Binh NG, Co DX, Tuan DQ, Ton MD, Dai KQ, Thach PT, Nagase H, Kudo K, Nguyen DA. Clinical epidemiology and mortality on patients with acute respiratory distress syndrome (ARDS) in Vietnam. PLoS One. 2019 Aug 15;14(8):e0221114. doi: 10.1371/journal.pone.0221114. PMID: 31415662; PMCID: PMC6695190.

Additional Editor Comments:

The study investigates factors associated with mortality in ARDS patients in a central hospital in Vietnam, analyzing inter-hospital care, clinical characteristics, and treatments that influence outcomes. Key findings include the protective role of endotracheal tube (ET) use during transport and the high mortality rate associated with high SOFA scores.

Our answer:

Thank you to the Editor and Reviewers for their support and for taking the time to provide their high-quality reviews.

Strengths

Provides a crucial analysis of ARDS in a lower middle-income context, filling a gap in ARDS research focused on LMICs.

Rigorous data collection and organization, with a comprehensive dataset that captures diverse patient characteristics, comorbidities, and treatment factors.

Use of multivariable logistic regression to isolate mortality predictors, enhancing the reliability of findings.

Our answer:

We appreciate your positive feedback. We have thoughtfully addressed the Editors' and Reviewers' comments and suggestions and revised our manuscript accordingly.

Weaknesses

Variable Selection Criteria in Regression: The logistic regression method would benefit from more detailed explanation. While significant variables were selected for

multivariable regression, a clear justification for each included variable would improve transparency, especially given potential multicollinearity.

Our answer:

Thank you for your valuable comment. We have provided a clear justification for each variable to be included in the multivariable logistic regression model. This justification is presented in the Statistical Analyses section and is also detailed below:

"(b) variables with a P-value of less than 0.25 in the univariable analysis comparing death and survival in the hospital, as well as those deemed clinically significant, were included in the multivariable logistic regression model. These variables, likely to influence patient outcomes, included inter-hospital care (e.g., MV applied in prior hospitalization, inter-hospital transport, inter-hospital care provider, inter-hospital airway, and inter-hospital oxygen), demographics (e.g., age, gender), documented comorbidities (i.e., chronic cardiac failure, active neoplasm, chronic renal failure, hematological disease, and CCI Score), gas exchange (e.g., PaO_2/FiO_2 ratio), the severity of illness (e.g., either SOFA Score or SOFA Score ≥cut-off value), adjunctive therapies (e.g., recruitment maneuvers, corticosteroids, ECAT, and tracheostomy), and complications (i.e., HAP, secondary bacterial infections, and septic shock)." (Lines 302-308, Pages 13-14)

Data Exclusions: The exclusion of patients with incomplete data could introduce selection bias. It would be beneficial to include an analysis of excluded cases or a sensitivity analysis to assess the impact of these exclusions on outcomes.

Our answer:

Thank you for your insightful comment. The Reviewer correctly noted that excluding patients with incomplete data might introduce selection bias. However, the excluded patients had missing data for most variables, making their inclusion in the analysis impossible. We have acknowledged this limitation in the Limitations section and is also detailed below:

"*Firstly*, its retrospective design restricted the availability of data for many variables (S8 Table as shown in S1 File). For instance, we only had information on ET usage during transportation for 193 patients. Additionally, many excluded patients had missing data for

most variables, making their inclusion in the analysis impossible. Excluding patients with incomplete data might also introduce selection bias." (Lines 547-551, Pages 31-32)

Inter-hospital Transport Analysis: The manuscript mentions transport categories (EMS, hospital ambulances, private, and public). However, a breakdown of patient outcomes by transport type, adjusted for severity and other covariates, would provide more actionable insights.

Our answer:

Thank you for your valuable comment. We have included a breakdown of patient outcomes by transport type in the S1 Table as shown in S1 File. However, we only had transport-type information for 214 out of 353 patients. As a result, we could not adjust the breakdown of patient outcomes by transport type for severity and other covariates in this dataset. We have acknowledged this limitation in the Limitations section and is also detailed below:

"*Thirdly*, the underdeveloped pre-hospital and inter-hospital transfer system prevents the integration of treatment protocols across pre-hospital, inter-hospital, and in-hospital settings in Vietnam.(30, 33, 60, 61) This issue also impacts the ability to collect clinical data for surveillance, quality improvement, and research purposes. Consequently, our study lacks detailed information on response times and quantitative benchmarks for each type of transport, including the minimum oxygen saturation levels required to evaluate the necessity for MV during transportation." (Lines 558-565, Page 32)

References

[30] Do SN, Luong CQ, Pham DT, Nguyen CV, Ton TT, Pham TTN, et al. Survival after out-of-hospital cardiac arrest, Viet Nam: multicentre prospective cohort study. Bulletin of the World Health Organization. 2021;99(1):50-61.

[33] Do SN, Luong CQ, Pham DT, Nguyen MH, Ton TT, Hoang QTA, et al. Survival after traumatic out-of-hospital cardiac arrest in Vietnam: a multicenter prospective cohort study. BMC emergency medicine. 2021;21(1):148.

[60] Xuan Dao C, Quoc Luong C, Manabe T, Ha Nguyen M, Thi Pham D, Thanh Ton T, et al. Impact of Bystander Cardiopulmonary Resuscitation on Out-of-Hospital Cardiac Arrest Outcome in Vietnam. The western journal of emergency medicine. 2024;25(4):507-20.

[61] Hoang BH, Mai TH, Dinh TS, Nguyen T, Dang TA, Le VC, et al. Unmet Need for Emergency Medical Services in Hanoi, Vietnam. JMA Journal. 2021;4(3):277-80.

Sample Size Justification: While the calculation is given, it lacks consideration of covariates in the multivariable analysis. A power analysis that accounts for expected covariate interactions in logistic regression could better support the robustness of findings.

Our answer:

Thank you for your valuable comment. In this study, we calculated the sample size, with consideration of covariates in the multivariable analysis. Specifically, we used a formula to determine the minimum sample size for estimating a population proportion with a 95% confidence level, a margin of error of ±5.21%, and an assumed population proportion of 47.8% based on the hospital mortality rate reported in a previous study [1]. Consequently, our sample size should be at least 353 patients, which is likely sufficient to reflect a normal distribution.

A well-known guideline suggests that the minimum required sample size should be based on the rule of events per variable (EPV) [2]. According to this rule, we needed to estimate the proportion of the outcome in the least category and divide it by 10 to determine the number of independent variables that could be studied. However, the concept of EPV with 10 has faced criticism [3], leading Austin and Steyerberg to recommend an EPV of 20 instead [4]. In our study, we had a sample size of 353 patients and considered 21 covariates in the multivariable analysis, complying with the EPV concepts of both 10 [3] and 20 [4].

Nevertheless, Nemes and colleagues, in their simulation study, demonstrated that a larger sample size, preferably 500, would increase the accuracy of the estimates [5]. A recent study also suggests that a minimum sample size of 500 is necessary to derive statistics that accurately represent the parameters in the target population.[6] In other words, the rules of thumb are an EPV of 50 and the formula $n = 100 + 50i$, where i refers to the number of independent variables in the final model. However, a sample size of less than 500 may be sufficient for associations that yield medium to large effect sizes.[6] Therefore, we have acknowledged this limitation in the Limitation section (Lines 565-573, Page 32).

References:

[1] Villar J, Blanco J, Añón JM, Santos-Bouza A, Blanch L, Ambrós A, Gandía F, Carriedo D, Mosteiro F, Basaldúa S, Fernández RL, Kacmarek RM; ALIEN Network. The ALIEN study: incidence and outcome of acute respiratory distress syndrome in the era of lung protective ventilation. Intensive Care Med. 2011 Dec;37(12):1932-41.

[2] Peduzzi P, Concato J, Kemper E, Holford TR, Feinstein AR. A simulation study of the number of events per variable in logistic regression analysis. J Clin Epidemiol. 1996 Dec;49(12):1373-9.

[3] van Smeden M, de Groot JA, Moons KG, Collins GS, Altman DG, Eijkemans MJ, Reitsma JB. No rationale for 1 variable per 10 events criterion for binary logistic regression analysis. BMC Med Res Methodol. 2016 Nov 24;16(1):163.

[4] Austin PC, Steyerberg EW. Events per variable (EPV) and the relative performance of different strategies for estimating the out-of-sample validity of logistic regression models. Stat Methods Med Res. 2017 Apr;26(2):796-808.

[5] Nemes S, Jonasson JM, Genell A, Steineck G. Bias in odds ratios by logistic regression modelling and sample size. BMC Med Res Methodol. 2009 Jul 27;9:56.

[6] Bujang MA, Sa'at N, Sidik TMITAB, Joo LC. Sample Size Guidelines for Logistic Regression from Observational Studies with Large Population: Emphasis on the Accuracy Between Statistics and Parameters Based on Real Life Clinical Data. Malays J Med Sci. 2018 Jul;25(4):122-130.

Smoking and Comorbidities Analysis: Smoking status is a significant factor in respiratory outcomes, yet its impact on ARDS outcomes in this cohort is not deeply analyzed. Similarly, the Charlson Comorbidity Index (CCI) score is included, but specific comorbidities could be separately analyzed for their mortality impact.

Our answer:

Thank you for your valuable comment. The Reviewer correctly identified that smoking status and specific comorbidities are significant factors in patient outcomes. In response, we

conducted additional analyses to assess the impact of these factors. However, our univariable and multivariable logistic regression analyses revealed that neither smoking status nor specific comorbidities were associated with hospital mortality. These findings are detailed in Table 7 (Pages 25-26).

Line-Specific Comments

Line 67 (Abstract, Background): To increase clarity, consider specifying which "factors associated with acute illness" are commonly associated with ARDS mortality. This would give readers immediate insight into whether these factors pertain to patient demographics, treatment variables, or environmental conditions.

Our answer:

Thank you for your valuable comment. We have clarified the factors commonly associated with acute respiratory distress syndrome (ARDS) mortality in low- and middle-income countries (LMICs) in the Abstract, which is also provided below:

"Acute respiratory distress syndrome (ARDS) has a high mortality rate, particularly in low- and middle-income countries, where the quality of pre-hospital or inter-hospital care can significantly impact patient outcomes. This study aimed to investigate mortality rates and associated factors in ARDS patients in Vietnam." (Lines 69-72, Page 4)

Line 89 (Abstract, Results): The interpretation of the odds ratio for endotracheal tube (ET) usage is crucial. Since a low OR (0.057) is unusual, clarify its significance by discussing possible mechanisms or logistical practices that could influence this protective effect.

Our answer:

Thank you for your valuable comment. To better interpret the adjusted odds ratio (OR) for endotracheal tube (ET) usage as a protective factor and discuss logistical practices to enhance this effect, we have clarified the adjusted OR for ET usage in the Results section of the Abstract. Additionally, we have included a recommendation on improving critical

interventions during transportation in the Conclusions section of the Abstract, as provided below:

"The univariable logistic regression identified several factors significantly associated with hospital mortality, including age (OR: 1.027; 95% CI: 1.013-1.040; p<0.001), PaO2/FiO2 ratio (OR: 0.993; 95% CI: 0.989-0.996; p<0.001), SOFA Score (OR: 1.168; 95% CI: 1.093-1.250; p<0.001), and septic shock (OR: 2.077; 95% CI: 1.338-3.226; p=0.001). However, the multivariable analysis revealed that only utilizing an ET during transportation was a protective factor against hospital mortality (adjusted OR: 0.070; 95% CI: 0.005-0.937; p=0.045)." (Lines 88-94, Pages 4-5)

"Utilizing ET during transportation emerged as a protective factor associated with a decreased risk of death. To reduce mortality in ARDS patients, the management of ARDS in Vietnam should focus on enhancing critical interventions during transportation, such as intubation and ventilation." (Lines 98-101, Page 5)

Line 112 (Introduction): The phrase "remains associated with high mortality rates" is broad. To strengthen this section, consider including recent global statistics or meta-analysis findings on ARDS mortality to contextualize Vietnam's figures in a broader global health framework.

Our answer:

Thank you for your comment. We have included data on ARDS mortality in the Introduction section, extracted from recent global statistics. These data are also provided below:

"Despite advances in the care of critically ill patients, mortality rates for those with ARDS remain alarmingly high, ranging from 26% to 60%(6-11). These rates vary across different institutions and countries, influenced by many confounding factors such as patient cohort characteristics, types of mortality reported, comorbidities, treatment strategies, and disease severity. A multicenter, international cohort study of 3022 patients with ARDS provides the best estimates, indicating an overall rate of death in the hospital of approximately 40.1% (952/2377).(9)" (Lines 114-120, Page 6)

References:

[6] Villar J, Blanco J, Añón JM, Santos-Bouza A, Blanch L, Ambrós A, Gandía F, Carriedo D, Mosteiro F, Basaldúa S, Fernández RL, Kacmarek RM; ALIEN Network. The ALIEN study: incidence and outcome of acute respiratory distress syndrome in the era of lung protective ventilation. Intensive Care Med. 2011 Dec;37(12):1932-41.

[7] Esteban A, Frutos-Vivar F, Muriel A, Ferguson ND, Peñuelas O, Abraira V, Raymondos K, Rios F, Nin N, Apezteguía C, Violi DA, Thille AW, Brochard L, González M, Villagomez AJ, Hurtado J, Davies AR, Du B, Maggiore SM, Pelosi P, Soto L, Tomicic V, D'Empaire G, Matamis D, Abroug F, Moreno RP, Soares MA, Arabi Y, Sandi F, Jibaja M, Amin P, Koh Y, Kuiper MA, Bülow HH, Zeggwagh AA, Anzueto A. Evolution of mortality over time in patients receiving mechanical ventilation. Am J Respir Crit Care Med. 2013 Jul 15;188(2):220-30.

[8] Wang CY, Calfee CS, Paul DW, Janz DR, May AK, Zhuo H, Bernard GR, Matthay MA, Ware LB, Kangelaris KN. One-year mortality and predictors of death among hospital survivors of acute respiratory distress syndrome. Intensive Care Med. 2014 Mar;40(3):388-96.

[9] Bellani G, Laffey JG, Pham T, Fan E, Brochard L, Esteban A, Gattinoni L, van Haren F, Larsson A, McAuley DF, Ranieri M, Rubenfeld G, Thompson BT, Wrigge H, Slutsky AS, Pesenti A; LUNG SAFE Investigators; ESICM Trials Group. Epidemiology, Patterns of Care, and Mortality for Patients With Acute Respiratory Distress Syndrome in Intensive Care Units in 50 Countries. JAMA. 2016 Feb 23;315(8):788-800.

[10] Pisani L, Algera AG, Serpa Neto A, Ahsan A, Beane A, Chittawatanarat K, Faiz A, Haniffa R, Hashemian SM, Hashmi M, Imad HA, Indraratna K, Iyer S, Kayastha G, Krishna B, Ling TL, Moosa H, Nadjm B, Pattnaik R, Sampath S, Thwaites L, Tun NN, Mohd Yunos N, Grasso S, Paulus F, Gama de Abreu M, Pelosi P, Day N, White NJ, Dondorp AM, Schultz MJ; PRoVENT-iMiC Investigators, MORU, and the PROVE Network. Epidemiological Characteristics, Ventilator Management, and Clinical Outcome in Patients Receiving Invasive Ventilation in Intensive Care Units from 10 Asian Middle-Income Countries (PRoVENT-iMiC): An International, Multicenter, Prospective Study. Am J Trop Med Hyg. 2021 Jan 11;104(3):1022-1033.

[11] Santa Cruz R, Matesa A, Gómez A, Nadur J, Pagano F, Prieto D, Bolaños O, Solis B, Yusta S, González-Velásquez E, Estenssoro E, Cavalcanti A. Mortality Due to Acute

Respiratory Distress Syndrome in Latin America. Crit Care Med. 2024 Aug 1;52(8):1275-1284.

Line 127 (Introduction): The introduction would benefit from a more in-depth explanation of how Vietnam's healthcare infrastructure specifically impacts ARDS outcomes, particularly regarding referral systems and delays in critical care.

Our answer:

Thank you for your comment. We have further explained how Vietnam's healthcare infrastructure specifically impacts ARDS outcomes, particularly regarding referral systems and delays in critical care, in the Introduction section (Lines 130-148, Pages 6-7). This topic is also discussed in detail in the Discussion section (Lines 443-507, Pages 27-30).

Line 168 (Methods, Patient Transport): The categories for patient transport are defined well, but it may improve the study's applicability if you provide more details on the quality or typical response time associated with each type of transport. This additional detail could help distinguish the types' impact on patient outcomes.

Our answer:

Thank you for your valuable comment. In Vietnam, the underdeveloped pre- and inter-hospital transfer system hinders the integration of treatment protocols across the pre-hospital, inter-hospital, and hospital settings and the collection of clinical data for surveillance, quality improvement, and research activities. As a result, time-stamped data for each type of transport are not available in our study. However, we have provided more details on the quality of care associated with each transport type in the Methods section, which are also included below:

"Hospital ambulances and emergency medical services are staffed by trained and accredited medical personnel. These services are equipped with life-saving equipment, operate under medical oversight, and have regularly monitored quality indicators. In contrast, private ambulance services provide emergency transportation with limited medical interventions

during transit. Patients arriving by private or public transport typically receive no first aid before reaching the hospital." (Lines 185-190, Page 9)

Line 201 (Variables): When discussing inter-hospital care data, consider adding any quantitative thresholds or benchmarks (e.g., minimum levels of oxygen saturation that determine MV application). This addition would provide context on how Vietnamese hospitals make critical care decisions during transfers.

Our answer:

Thank you for your valuable comment. In Vietnam, the underdeveloped pre- and inter-hospital transfer system hinders the integration of treatment protocols across the pre-hospital, inter-hospital, and hospital settings and the collection of clinical data for surveillance, quality improvement, and research activities. As a result, our study lacks detailed data on quantitative thresholds or benchmarks, such as the minimum levels of oxygen saturation required to determine the application of mechanical ventilation (MV) for each type of transport. We have addressed this limitation in the Limitations section and is also detailed below:

"*Thirdly*, the underdeveloped pre-hospital and inter-hospital transfer system prevents the integration of treatment protocols across pre-hospital, inter-hospital, and in-hospital settings in Vietnam.(30, 33, 60, 61) This issue also impacts the ability to collect clinical data for surveillance, quality improvement, and research purposes. Consequently, our study lacks detailed information on response times and quantitative benchmarks for each type of transport, including the minimum oxygen saturation levels required to evaluate the necessity for MV during transportation." (Lines 558-565, Page 32)

References

[30] Do SN, Luong CQ, Pham DT, Nguyen CV, Ton TT, Pham TTN, et al. Survival after out-of-hospital cardiac arrest, Viet Nam: multicentre prospective cohort study. Bulletin of the World Health Organization. 2021;99(1):50-61.

[33] Do SN, Luong CQ, Pham DT, Nguyen MH, Ton TT, Hoang QTA, et al. Survival after traumatic out-of-hospital cardiac arrest in Vietnam: a multicenter prospective cohort study. BMC emergency medicine. 2021;21(1):148.

[60] Xuan Dao C, Quoc Luong C, Manabe T, Ha Nguyen M, Thi Pham D, Thanh Ton T, et al. Impact of Bystander Cardiopulmonary Resuscitation on Out-of-Hospital Cardiac Arrest Outcome in Vietnam. The western journal of emergency medicine. 2024;25(4):507-20.

[61] Hoang BH, Mai TH, Dinh TS, Nguyen T, Dang TA, Le VC, et al. Unmet Need for Emergency Medical Services in Hanoi, Vietnam. JMA Journal. 2021;4(3):277-80.

Line 256 (Outcomes): While hospital mortality is the primary outcome, detailing any standardized discharge criteria could clarify how patients' outcomes are assessed upon transfer or discharge, helping to interpret mortality rates across different levels of care.

Our answer:

Thank you for your comment. This study included only hospital mortality data extracted from medical records. At our study site, the decision to transfer or discharge ARDS patients is at the attending physician's discretion, who considers bed availability and overall hospital capacity. These factors are detailed below:

"The primary outcome measured was hospital mortality, defined as death from any cause during hospitalization. At this study site, the attending physician decides whether to transfer or discharge ARDS patients, considering bed availability and overall hospital capacity. We also examined secondary outcomes, including complications and hospital length of stay (LOS)." (Lines 270-274, Page 12)

Line 271 (Statistical Analyses): The analysis methods are detailed, but the selection criteria for variables in the logistic regression model could be more precise. Mentioning any variable selection methods (like stepwise selection or feature importance ranking) would enhance the rigor of this section.

Our answer:

Thank you for your valuable comment. We have provided a clear justification for each variable to be included in the multivariable logistic regression model. This justification is presented in the Statistical Analyses section (Lines 296-315, Pages 13-14)

Line 315 (Results, Data Exclusions): Excluding cases with missing data may introduce bias, especially if those cases had particularly severe or mild conditions. Discussing potential implications of these exclusions would improve transparency.

Our answer:

Thank you for your valuable comment. We have elaborated on this limitation in the Limitations section and is also detailed below:

"*Firstly*, its retrospective design restricted the availability of data for many variables (S8 Table as shown in S1 File). For instance, we only had information on ET usage during transportation for 193 patients. Additionally, many excluded patients had missing data for most variables, making their inclusion in the analysis impossible. Excluding patients with incomplete data might also introduce selection bias." (Lines 547-551, Pages 31-32)

Line 346 (Results, Smoking Habits): Since smoking is a significant factor for respiratory distress, a breakdown of its impact on ARDS severity or mortality would add depth to the study findings. Consider performing an analysis to examine if there's an association between smoking status and mortality outcomes.

Our answer:

Thank you for your valuable comment. The Reviewer correctly identified that smoking status and specific comorbidities are significant factors in patient outcomes. In response, we conducted additional analyses to assess the impact of these factors. However, our univariable logistic regression analyses revealed that neither smoking status nor specific comorbidities were associated with hospital mortality. These findings are detailed in the following Table.

To identify independent predictors of hospital mortality, we selected variables with a P-value of less than 0.25 from the univariable analysis comparing hospital deaths and survivals, as well as those deemed clinically significant, for inclusion in the multivariable logistic regression model. These variables included specific comorbidities (i.e., chronic cardiac failure, active neoplasm, chronic renal failure, hematological disease), the CCI Score, and 16 other independent variables. Consequently, we found that only the presence of an

endotracheal tube, a SOFA Score ≥ 9.5, and ECAT remained independently associated with hospital mortality (Table 7 and S7 Table in S1 File).

Table 1. Association of habitual behavior and comorbidities with hospital mortality in patients with acute respiratory distress syndrome: univariable logistic regression analyses

Factor	OR	95.0% CI for OR		p-value
		Lower	Upper	
**Habitual behavior**				
Smoking history				
Never	Reference	-	-	0.390
Quit	1.787	0.738	4.329	0.198
Current	0.958	0.517	1.770	0.887
**Comorbidities**				
Cerebrovascular disease	1043488548.766927	0.000	-	0.999
Chronic cardiac failure, no. (%)	2.310	0.479	11.138	0.297
Coronary artery disease/ myocardial infarction	918147584.755691	0.000	-	0.999
Hypertension	0.955	0.529	1.725	0.879
Chronic obstructive pulmonary disease/asthma	1023545143.040535	0.000	-	0.999
Other chronic pulmonary disease	0.770	0.349	1.700	0.518
Tuberculosis	2.343	0.257	21.318	0.450
Active neoplasm	2.700	0.569	12.807	0.221
Chronic renal failure	0.526	0.217	1.271	0.153
Ulcer disease	1.266	0.374	4.287	0.705
Diabetes mellitus	1.056	0.535	2.086	0.875
Immunoincompetence, no. (%)	0.736	0.264	2.053	0.558
Hematological disease	1.936	0.611	6.137	0.262
Charlson Comorbidity index	1.086	0.914	1.290	0.347

Line 375 (Results, Table 4 - PaO2/FiO2 Ratio): The PaO2/FiO2 ratio is presented in both text and table form. To streamline, a graphical representation of these ratios with survival and mortality outcomes might improve interpretability, as this metric is a core component of the Berlin definition for ARDS severity.

Our answer:

Thank you for your comment. We only have data on the PaO_2/FiO_2 ratio upon admission. Therefore, we presented it in table form for better clarity and understanding.

Line 403 (Table 7, Multivariable Regression): Given the high odds ratio for SOFA score, it could be beneficial to mention specific SOFA components (e.g., respiratory or cardiovascular factors) that contribute most significantly to this outcome. This would highlight which organ systems might be prioritized in treatment protocols.

Our answer:

Thank you for your insightful comment. We conducted univariable logistic regression analyses to examine the association between hospital mortality and specific SOFA components, including the PaO_2/FiO_2 ratio, platelet count, bilirubin levels, blood pressure or vasoactive agent administration (i.e., septic shock), Glasgow coma scale score, and creatinine levels. Our findings revealed that only the PaO_2/FiO_2 ratio and the presence of septic shock were significantly associated with an increased risk of hospital mortality in the univariable logistic regression analyses (Table 7). Consequently, we incorporated these significant independent variables and 19 other independent variables into our multivariable logistic regression analysis (Table 7 and S7 Table in S1 File). Ultimately, we identified that only the presence of an endotracheal tube, a SOFA Score $\geq$ 9.5, and ECAT remained independently associated with hospital mortality (Table 7 and S7 Table in S1 File).

Minor Comments

Line-by-Line Edits: Some minor editorial adjustments for grammar and clarity are needed in the abstract and results sections.

Our answer:

Thank you for your comment. We have corrected grammatical errors and clarified sentences in the abstract and results sections.

Graphs and Tables: Visual aids, such as survival curves stratified by key risk factors (e.g., SOFA scores, PaO2/FiO2 ratios), would improve result interpretability.

Our answer:

Thank you for your comment. We only have data on the SOFA score and PaO_2/FiO_2 ratio upon admission. Consequently, we were unable to present survival curves stratified by these key risk factors.

Statistical Detail: Reporting effect sizes (e.g., odds ratios) with more precise confidence intervals in the abstract would better convey result certainty.

Our answer:

Thank you for your comment. We have already reported the effect sizes (i.e., odds ratios) with confidence intervals to measure the strength of the association between the independent variables (e.g., ET usage) and hospital mortality in both the Abstract (Lines 88-94, Pages 4-5) and the Results section (Lines 413-426, Page 24).

Thank the Editors and Reviewers so much for taking the time to leave their high-quality reviews.

Sincerely,

Chinh Quoc Luong, MD, PhD
Center for Emergency Medicine,
Bach Mai Hospital,
No. 78, Giai Phong, Dong Da district,

Hanoi 100000, Vietnam

Email: luongquocchinh@gmail.com

Son Ngoc Do, MD, PhD

Center for Critical Care Medicine,

Bach Mai Hospital,

No. 78, Giai Phong Road, Dong Da District,

Hanoi, 100000, Vietnam

Email: sonngocdo@gmail.com
