# Supplementary material for: Factors related to mortality in patients with acute respiratory distress syndrome (ARDS) in a lower middle-income country: A retrospective observational study
Source: PLoS One. 2025 Nov 18;20(11):e0337071. doi: 10.1371/journal.pone.0337071 (PMC12626306; doi:10.1371/journal.pone.0337071)
Supplement: Attachment — Submitted filename: Response_to_Reviewers_auresp_2.pdf [file pone.0337071.s004.pdf]

Dr. Gurmeet Singh, M.D., Ph.D.
Academic Editor
PLOS ONE
August 18, 2025

Dear Dr. Gurmeet Singh,

On behalf of all the authors, we are resubmitting our revised manuscript titled *"Factors related to mortality in patients with acute respiratory distress syndrome (ARDS) in a lower middle-income country: a retrospective observational study"* (PONE-D-24-35750R1).

We sincerely appreciate the thoughtful comments and suggestions provided by the Editors and Reviewers. We have carefully addressed all feedback and revised our manuscript accordingly. These insights have significantly improved the quality of our work, and we hope the Editor finds our revised manuscript suitable for publication in PLOS ONE.

We confirm that this research is original, has not been published elsewhere, and is not currently under consideration by any other journal. All authors have read and approved the manuscript, have agreed on the order of authorship, and hold the necessary permissions and rights for the data presented.

Below, we have included our point-by-point responses to the comments from the Editor and the Reviewers. Thank you for considering this submission.

Sincerely,

Chinh Quoc Luong, MD, PhD
Neuro Intensive Care Department,
Neurology Center, Bach Mai Hospital,
No. 78, Giai Phong, Dong Da district,
Hanoi 100000, Vietnam
Email: luongquocchinh@gmail.com

Son Ngoc Do, MD, PhD
Center for Critical Care Medicine,
Bach Mai Hospital,
No. 78, Giai Phong Road, Dong Da District,

Hanoi, 100000, Vietnam.
Email: sonngocdo@gmail.com

*****

We thank the Editors and the Reviewers for the valuable comments and suggestions that have helped us improve the contents of this paper. In what follows, we have used the **boldface** to indicate the Editors' and the Reviewers' comments and the standard font face for our responses. We also highlighted in yellow the modifications that we performed on the manuscript.

RESPONSE TO EDITOR COMMENTS

PONE-D-24-35750R1

Factors related to mortality in patients with acute respiratory distress syndrome (ARDS) in a lower middle-income country: a retrospective observational study

PLOS ONE

Dear Dr. Do,

Thank you for submitting your manuscript to PLOS ONE. After careful consideration, we feel that it has merit but does not fully meet PLOS ONE's publication criteria as it currently stands. Therefore, we invite you to submit a revised version of the manuscript that addresses the points raised during the review process.

Our answer:

Thank you for your encouraging feedback. We appreciate the time and effort the Editors and Reviewers have devoted to evaluating our manuscript. In response to the valuable comments and suggestions provided, we have carefully revised the manuscript to address all the points raised. We hope the changes made meet the journal's standards and improve the clarity and rigor of our study.

ACADEMIC EDITOR:

Thank you for submitting the revised manuscript titled Factors related to mortality in patients with acute respiratory distress syndrome (ARDS) in a lower-middle-income country: a retrospective observational study to Plos One. I appreciate the effort you have put into addressing the reviewers' comments, and the manuscript has improved significantly.

However, several concerns still need to be addressed before the manuscript can be considered for publication:

Our answer:

Thank you very much for your encouraging feedback and for recognizing the improvements made in our revised manuscript. We are sincerely grateful to you and the Reviewers for the time, effort, and thoughtful insights provided throughout the review process.

In response to the remaining concerns outlined in your latest comments, we have conducted another thorough revision of the manuscript. We carefully addressed each point raised, making the necessary changes to enhance the clarity, consistency, and scientific rigor of our work. These revisions include both substantive content updates and refinements to language and formatting, in line with the journal’s standards.

The manuscript is now significantly strengthened and better positioned to make a meaningful contribution to the literature on ARDS, particularly in the context of lower-middle-income countries. We hope the revised version meets your expectations. We are happy to provide any additional clarification or further revisions if needed.

Thank you again for your guidance and continued consideration of our work.

Key Concerns:

1. Duality of Publication:

- The manuscript presents an update to your previously published work. Please ensure that you clearly explain why this updated study does not constitute dual publication,

both in the cover letter and within the manuscript. This explanation should emphasize that the new data adds significant value and is not a mere repetition of the previous findings.

Our answer:

Thank you for your thoughtful comment regarding the potential for overlap with our previously published work. We appreciate the opportunity to clarify how the current manuscript represents a distinct and original contribution to the literature, rather than a duplicate publication.

While the current study builds upon our earlier research published in PLOS ONE [1], it significantly extends and deepens the scope of investigation in several key ways:

- Expanded Study Period: The previous study analyzed data from ARDS patients admitted to Bach Mai Hospital (BMH) between August 2015 and August 2017. In contrast, the current manuscript incorporates six additional years of data, covering admissions from September 2017 to August 2023. This extended timeframe enables a more comprehensive and representative analysis of ARDS trends and outcomes over nearly a decade.

- New Ethical Approval and Dataset Integration: The extended dataset was approved by the BMH Scientific and Ethics Committees on August 11, 2023. The original and new datasets were merged and reanalyzed using updated statistical methods, enabling a more comprehensive and nuanced understanding of ARDS in this setting.

- Novel Analytical Focus: Unlike the earlier study, which primarily described clinical characteristics and outcomes, the current manuscript introduces new variables and analytical dimensions, with a particular focus on transportation-related factors, pre-hospital care, and inter-hospital transfer practices. These aspects were not explored in the previous publication and are especially relevant in the context of healthcare delivery in lower-middle-income countries.

- New Findings and Interpretations: The present study yields several novel findings, including the independent association between endotracheal intubation during transport and reduced hospital mortality, as well as the impact of extracorporeal cytokine adsorption

therapy (ECAT) on outcomes. These insights provide added clinical and policy relevance that was not addressed in the earlier work.

Given these substantial additions and methodological enhancements, we respectfully submit that this manuscript constitutes a significant advancement beyond our prior publication. It offers new evidence and perspectives that are highly relevant to the global ARDS literature, particularly in resource-limited settings.

We have also included a brief explanation of this distinction in the revised manuscript (Pages 7-8, Lines 159-166) and will ensure that it is clearly stated in the cover letter as well.

Thank you again for your careful consideration.

[1] Chinh LQ, Manabe T, Son DN, Chi NV, Fujikura Y, Binh NG, Co DX, Tuan DQ, Ton MD, Dai KQ, Thach PT, Nagase H, Kudo K, Nguyen DA. Clinical epidemiology and mortality on patients with acute respiratory distress syndrome (ARDS) in Vietnam. PLoS One. 2019 Aug 15;14(8):e0221114. doi: 10.1371/journal.pone.0221114. PMID: 31415662; PMCID: PMC6695190.

2. Analysis of the Pandemic Period:

- The inclusion of data from the pandemic period (COVID-19) is important but currently lacks analysis of its impact on ARDS outcomes. I recommend performing a comparative analysis between the pre-pandemic and pandemic periods to provide a clearer understanding of how the pandemic may have influenced the results.

Our answer:

Thank you for your insightful comment regarding the inclusion of data from the COVID-19 pandemic period and its potential impact on ARDS outcomes. We fully agree that this is an important consideration, and we have addressed it explicitly in the revised manuscript.

In response, we conducted a subgroup analysis comparing patients admitted during the pre-pandemic period (August 2015 to December 2019) with those admitted during the pandemic period (January 2020 to August 2023). This analysis examined key clinical characteristics, management strategies, and hospital mortality rates across the two timeframes. While we

observed some differences in variables such as the use of non-invasive ventilation, rates of endotracheal intubation, and the prevalence of viral pneumonia (particularly influenza A), these differences did not reach statistical significance in terms of overall hospital mortality. However, the analysis revealed essential trends, including a shift in the etiological profile of ARDS and evolving patterns in respiratory support practices during the pandemic. These findings are now presented in the Results section (Page 26, Lines 449-464; Table 7, S6-S12 Tables as shown in S1 File), and a corresponding discussion (Pages 34-35, Lines 604-629) has been added to contextualize them within the broader literature on ARDS management during the COVID-19 pandemic. This comparative analysis enhances the manuscript by providing a clearer understanding of how the pandemic may have influenced ARDS outcomes in a lower-middle-income country setting. It also underscores the adaptability of clinical practices in response to emerging infectious threats and resource constraints.

Thank you again for this valuable suggestion, which we believe has meaningfully strengthened the manuscript's depth and relevance.

3. Methodological Clarifications:

- Please provide further clarification on how potential multicollinearity was assessed in the multivariable logistic regression analysis. This would increase confidence in the robustness of your statistical methods.

Our answer:

Thank you for your insightful comment. To ensure the robustness of our model, we evaluated multicollinearity using the Variance Inflation Factor (VIF) for each predictor included in the final model (Page 15, Lines 343–345). All VIF values were below the commonly accepted threshold of 5, indicating that multicollinearity was not a concern and supporting the stability and interpretability of the regression coefficients. Additional details are provided in the note box beneath Table 8 and under S14 Table within the S1 File.

- Consider including a sensitivity analysis or a more in-depth discussion on how the exclusion of patients with incomplete data might have influenced the results, especially regarding selection bias.

Our answer:

Thank you for your thoughtful and constructive feedback. We agree that methodological transparency is critical for ensuring the robustness and credibility of our findings.

In response, we have revised the Methods section to provide greater detail on the handling of incomplete data and its potential impact on our results. Specifically, patients with missing key variables required for multivariable analysis were excluded. We recognize that this exclusion may introduce selection bias, particularly if the missingness is not entirely at random. While we considered statistical approaches such as multiple imputation and sensitivity analyses, the extent and pattern of missing data, especially in variables central to our primary outcomes, limited the feasibility and reliability of these methods. To address this concern, we have now included a more in-depth discussion of the potential implications of this exclusion in the revised manuscript (Page 35, Lines 631-638). To assess the robustness of our model despite these limitations, we evaluated its performance using multiple diagnostic criteria, including the Hosmer-Lemeshow goodness-of-fit test, pseudo-R^2 values, and log-likelihood statistics (Page 15, Lines 345–347). These metrics indicated acceptable calibration and discrimination. Additional details are provided in the note box beneath Table 8 and under S14 Table within the S1 File.

We hope these revisions adequately address your concerns and strengthen the transparency of our analytical approach.

4. Inter-hospital Transport Analysis:

- A breakdown of patient outcomes by transport type (e.g., EMS, hospital ambulances, private, and public) would provide more actionable insights. While data limitations are acknowledged, please expand on how the lack of detailed transport data impacts the analysis and its conclusions.

Our answer:

Thank you for your insightful and constructive comment regarding the analysis of inter-hospital transport. A more granular breakdown of patient outcomes by transport type (e.g., EMS, hospital-based ambulances, private vehicles, or public transport) would offer valuable insights into the role of pre-hospital care in ARDS outcomes.

Unfortunately, due to the retrospective design of our study and limitations in medical record documentation, detailed information on the specific mode of transport was not consistently available. Among the 353 patients included in the study, transport data were available for only 214 individuals. Even within this subset, the classification of transport type was often incomplete or inconsistently recorded, precluding a reliable stratified analysis by transport modality. We acknowledge that this limitation may impact the depth of our conclusions regarding the effect of inter-hospital transport on patient outcomes. Specifically, the absence of detailed transport data may have introduced selection bias in our multivariable logistic regression model, potentially leading to the underrepresentation of important pre-hospital factors such as transport duration, level of care during transfer, and type of vehicle used. These variables may influence both the severity of illness upon arrival and the subsequent risk of mortality. To address this limitation, we have expanded the Limitations section of the revised manuscript to explicitly acknowledge the potential impact of missing transport data on the robustness of our findings (Page 35, Lines 632-638). Despite these constraints, our analysis still revealed a critical and actionable finding: the use of endotracheal intubation during transport was independently associated with reduced hospital mortality, underscoring the importance of ensuring adequate respiratory support during inter-facility transfers, regardless of the type of transport.

We appreciate the opportunity to clarify this aspect of our study and believe that the revised discussion more accurately reflects both the strengths and limitations of our transport-related findings.

5. Cytokine Adsorption Therapy:

- Include a brief discussion on the potential impact of cytokine adsorption therapy on outcomes. Even if no formal analysis was done, acknowledging its role and the limitations of this therapy would add valuable context.

Our answer:

Thank you very much for your thoughtful and constructive suggestion. A brief discussion of the potential impact of cytokine adsorption therapy (ECAT) would provide essential context, even in the absence of a formal efficacy analysis.

In response, we have revised and expanded the Discussion section to include a more nuanced interpretation of the role of ECAT in ARDS management and its limitations within our study. The revised text now reads:

"ARDS is increasingly recognized as a syndrome of systemic immune dysregulation. Although the concept of a cytokine storm was initially suggested,(76) later studies have indicated that systemic cytokine levels in ARDS patients are generally lower than previously thought, except in severe COVID-19 cases(26, 77). The use of ECAT has been explored as a potential intervention for immune modulation. While detailed data on ECAT were not available in this study, the use of ECAT was associated with an increased risk of hospital mortality (S14 Table as shown in S1 File), likely reflecting its role as a rescue therapy for critically ill patients (S15 Table as shown in S1 File). This pattern suggests confounding by indication rather than a direct harmful effect. Given the observational nature of our study, definitive conclusions regarding the efficacy or safety of ECAT must be validated through prospective studies." (Pages 34, Lines 591-601)

This expanded discussion provides a more balanced and informative perspective on ECAT, aligning with your recommendation to contextualize its use and limitations. Thank you again for your helpful suggestion.

6. Minor Language and Formatting Edits:

- Please ensure the manuscript is carefully proofread for minor language issues, particularly with the consistency of abbreviations and proper article usage.

Our answer:

We sincerely thank the Editor for the helpful and constructive feedback regarding language and formatting. In response, we have conducted a thorough and careful proofreading of the entire manuscript to address all minor language issues and improve overall readability.

Specifically, we have:

- Ensured consistent and correct use of abbreviations throughout the manuscript. All abbreviations are now clearly defined at first mention and used uniformly thereafter (e.g., “endotracheal tube (ET)” is consistently abbreviated as “ET” following its initial definition).

- Reviewed and corrected article usage (e.g., “a,” “an,” “the”) to improve grammatical accuracy and sentence flow.

- Refined sentence structure and word choice in several sections to enhance clarity, precision, and readability for a broad audience.

- Standardized formatting in tables, figure legends, and references to align with the journal’s style guidelines.

We believe these revisions have improved the manuscript’s polish and presentation, and we appreciate the Editor’s attention to detail in helping us strengthen the quality of our submission.

Conclusion:

Your study presents valuable insights into the mortality factors for ARDS patients in a resource-limited setting. To move forward with publication, the manuscript requires further clarification regarding the pandemic data analysis, dual publication concerns, and some methodological details.

I recommend that you submit a major revision addressing these points. Once these revisions are made, the manuscript will be reconsidered for final publication.

Our answer:

We sincerely thank the Editor for the thoughtful, constructive, and encouraging feedback. We truly appreciate the opportunity to revise and enhance our manuscript in response to your

recommendations. We have carefully and comprehensively addressed each of the key points raised to improve the clarity, methodological rigor, and overall impact of the study.

The revisions made have substantially strengthened the manuscript and effectively resolved the concerns outlined. We are grateful for your guidance throughout the review process and for the opportunity to submit this major revision. We respectfully hope that the revised version meets the journal's standards and will be favorably considered for final publication.

Please submit your revised manuscript by Jul 30 2025 11:59PM. If you will need more time than this to complete your revisions, please reply to this message or contact the journal office at plosone@plos.org. When you're ready to submit your revision, log on to https://www.editorialmanager.com/pone/ and select the 'Submissions Needing Revision' folder to locate your manuscript file.

Our answer:

Thank you for the reminder regarding the submission deadline. We apologize for the delay and appreciate your patience. The revised manuscript has now been completed and submitted through the Editorial Manager system. We hope it can still be considered for review.

Please include the following items when submitting your revised manuscript:

- **A rebuttal letter that responds to each point raised by the academic editor and reviewer(s). You should upload this letter as a separate file labeled 'Response to Reviewers'.**
- **A marked-up copy of your manuscript that highlights changes made to the original version. You should upload this as a separate file labeled 'Revised Manuscript with Track Changes'.**
- **An unmarked version of your revised paper without tracked changes. You should upload this as a separate file labeled 'Manuscript'.**

Our answer:

Thank you for your guidance. In line with the journal's submission requirements, we have included the following documents with our revised manuscript: (1) a rebuttal letter responding to each comment from the academic editor and reviewers, (2) a marked-up version of the manuscript highlighting all revisions, and (3) a clean, unmarked version of the revised manuscript.

If you would like to make changes to your financial disclosure, please include your updated statement in your cover letter. Guidelines for resubmitting your figure files are available below the reviewer comments at the end of this letter.

Our answer:

Thank you for the guidance. We have reviewed our financial disclosure and confirm that no changes are necessary at this time. Accordingly, the original financial disclosure statement remains accurate and has been retained in the revised submission. We appreciate the clarification regarding the figure file resubmission and have followed the provided guidelines to ensure compliance with journal requirements.

If applicable, we recommend that you deposit your laboratory protocols in protocols.io to enhance the reproducibility of your results. Protocols.io assigns your protocol its own identifier (DOI) so that it can be cited independently in the future.

For instructions see:
https://journals.plos.org/plosone/s/submission-guidelines#loc-laboratory-protocols.

Additionally, PLOS ONE offers an option for publishing peer-reviewed Lab Protocol articles, which describe protocols hosted on protocols.io. Read more information on sharing protocols at:
https://plos.org/protocols?utm_medium=editorial-email&utm_source=authorletters&utm_campaign=protocols.

Our answer:

Thank you for the helpful suggestion. We recognize the importance of promoting transparency and reproducibility by sharing detailed protocols. However, our study did not involve laboratory-based procedures; therefore, laboratory protocols do not apply to our research. Instead, our study employed a retrospective observational design, and the corresponding study protocol is clearly described in the Methods section of the revised manuscript. We hope this approach sufficiently conveys the methodological framework of our research. Please let us know if any additional documentation would be beneficial.

We look forward to receiving your revised manuscript.

Kind regards,

Gurmeet Singh, M.D., Ph.D.,
Academic Editor
PLOS ONE

Our answer:

We sincerely thank the Academic Editor, Dr. Gurmeet Singh, and the Reviewers for their thoughtful and constructive feedback. We truly appreciate the time and effort invested in evaluating our manuscript and providing high-quality reviews. Their insights have been invaluable in helping us improve the clarity and quality of our work. We have carefully addressed all comments and look forward to the continued editorial evaluation of our revised submission.

RESPONSE TO REVIEWER COMMENTS

Review Comments to the Author

Reviewer #1:

I am very concerned about duality of publication

Our answer:

Thank you for your thoughtful comment regarding the potential for overlap with our previously published work. We appreciate the opportunity to clarify how the current manuscript represents a distinct and original contribution to the literature, rather than a duplicate publication.

While the current study builds upon our earlier research published in PLOS ONE [1], it significantly extends and deepens the scope of investigation in several key ways:

- Expanded Study Period: The previous study analyzed data from ARDS patients admitted to Bach Mai Hospital (BMH) between August 2015 and August 2017. In contrast, the current manuscript incorporates six additional years of data, covering admissions from September 2017 to August 2023. This extended timeframe enables a more comprehensive and representative analysis of ARDS trends and outcomes over nearly a decade.

- New Ethical Approval and Dataset Integration: The extended dataset was approved by the BMH Scientific and Ethics Committees on August 11, 2023. The original and new datasets were merged and reanalyzed using updated statistical methods, enabling a more comprehensive and nuanced understanding of ARDS in this setting.

- Novel Analytical Focus: Unlike the earlier study, which primarily described clinical characteristics and outcomes, the current manuscript introduces new variables and analytical dimensions, with a particular focus on transportation-related factors, pre-hospital care, and inter-hospital transfer practices. These aspects were not explored in the previous publication and are especially relevant in the context of healthcare delivery in lower-middle-income countries.

- New Findings and Interpretations: The present study yields several novel findings, including the independent association between endotracheal intubation during transport and reduced hospital mortality, as well as the impact of extracorporeal cytokine adsorption therapy (ECAT) on outcomes. These insights provide added clinical and policy relevance that was not addressed in the earlier work.

Given these substantial additions and methodological enhancements, we respectfully submit that this manuscript constitutes a significant advancement beyond our prior publication. It offers new evidence and perspectives that are highly relevant to the global ARDS literature, particularly in resource-limited settings.

We have also included a brief explanation of this distinction in the revised manuscript (Pages 7-8, Lines 159-166) and will ensure that it is clearly stated in the cover letter as well.

Thank you again for your careful consideration.

[1] Chinh LQ, Manabe T, Son DN, Chi NV, Fujikura Y, Binh NG, Co DX, Tuan DQ, Ton MD, Dai KQ, Thach PT, Nagase H, Kudo K, Nguyen DA. Clinical epidemiology and mortality on patients with acute respiratory distress syndrome (ARDS) in Vietnam. PLoS One. 2019 Aug 15;14(8):e0221114. doi: 10.1371/journal.pone.0221114. PMID: 31415662; PMCID: PMC6695190.

However, what I find more concerning is the inclusion of the period of the pandemic without analysis of the period to enrich the manuscript and perhaps make the question of duality even less of an issue.

Our answer:

Thank you for your insightful comment regarding the inclusion of data from the COVID-19 pandemic period and its potential impact on ARDS outcomes. We fully agree that this is an important consideration, and we have addressed it explicitly in the revised manuscript.

In response, we conducted a subgroup analysis comparing patients admitted during the pre-pandemic period (August 2015 to December 2019) with those admitted during the pandemic period (January 2020 to August 2023). This analysis examined key clinical characteristics, management strategies, and hospital mortality rates across the two timeframes. While we observed some differences in variables such as the use of non-invasive ventilation, rates of endotracheal intubation, and the prevalence of viral pneumonia (particularly influenza A), these differences did not reach statistical significance in terms of overall hospital mortality. However, the analysis revealed essential trends, including a shift in the etiological profile of ARDS and evolving patterns in respiratory support practices during the pandemic. These findings are now presented in the Results section (Page 26, Lines 449-464; Table 7, S6-S12 Tables as shown in S1 File), and a corresponding discussion (Pages 34-35, Lines 604-629) has been added to contextualize them within the broader literature on ARDS management during the COVID-19 pandemic. This comparative analysis enhances the manuscript by

providing a clearer understanding of how the pandemic may have influenced ARDS outcomes in a lower-middle-income country setting. It also underscores the adaptability of clinical practices in response to emerging infectious threats and resource constraints.

Thank you again for this valuable suggestion, which we believe has meaningfully strengthened the manuscript's depth and relevance.

Reviewer #2:

This study addresses an important clinical question about ARDS outcomes in a resource-limited setting. The large sample size (353 patients over 8 years) is a notable strength, providing more robust statistical power than many single-center studies. Data collection was thorough – using a standardized case report form and Berlin definition criteria ensures the patient population is well-defined

Our answer:

We sincerely appreciate your positive evaluation of our study. Your recognition of the clinical importance of investigating ARDS outcomes in a resource-limited setting, as well as the value of our extended dataset, is deeply encouraging.

As noted, the inclusion of 353 patients over eight years allows for a more statistically robust analysis compared to many previous single-center studies. We agree that using a standardized case report form, along with strict adherence to the Berlin definition criteria for ARDS, was essential in ensuring data consistency and the reliability of our findings.

We have carefully reviewed and integrated your comments into the revised manuscript to strengthen its clarity and rigor further. Your support reaffirms the relevance of this research in helping improve critical care outcomes in low- and middle-income countries.

The analysis is rigorous, combining univariate and multivariate methods to identify independent mortality factors. The finding that early airway management during transfer (endotracheal intubation) is associated with improved survival is particularly novel and has practical implications. The manuscript is also well-organized and

generally clear, which helps in conveying the findings. Overall, the research appears carefully conducted and fills a knowledge gap regarding pre-hospital care impact on ARDS outcomes in lower-middle-income countries.

Our answer:

We sincerely appreciate your thoughtful and encouraging review of our manuscript. We are particularly grateful for your recognition of the study's methodological rigor, including the use of both univariate and multivariate analyses to identify independent predictors of mortality in ARDS patients.

Your observation regarding the novel finding—that early airway management during patient transfer, specifically endotracheal intubation, was associated with improved survival—is especially gratifying. We agree that this insight has important clinical implications, particularly in resource-limited settings where pre-hospital care is often limited or under-optimized. By highlighting this association, our study aimed to inform evidence-based improvements in transport protocols and early intervention strategies in lower-middle-income countries.

We also appreciate your comments on the manuscript's organization and clarity. In response, we have carefully reviewed the text to ensure that the findings are presented as clearly and as easily as possible for a broad readership.

Once again, thank you for your valuable feedback. Your remarks reaffirm the importance and practical relevance of our work in advancing ARDS care and informing healthcare delivery in resource-limited settings.

Suggestions for Improvement:

Clarify the multivariable model building: Please add a brief description of how variables were chosen for the multivariate logistic regression (e.g. based on p-value cutoff or clinical importance). This will assure readers that the model was constructed without bias.

Our answer:

Thank you for your thoughtful and constructive comment. We agree that transparent reporting of variable selection is essential for ensuring methodological rigor and minimizing bias. To develop the multivariable logistic regression model, we used a structured approach that combined statistical criteria with clinical relevance. We initially screened variables through univariable logistic regression analyses, and those with a p-value <0.25 were considered candidates for multivariable modeling, consistent with established practices to avoid excluding potentially essential predictors. Additionally, variables deemed clinically significant—based on prior literature and expert consensus—were included regardless of their univariable p-values (Pages 14-15, Lines 307-331). We performed model refinement using stepwise backward elimination, beginning with all candidate variables. Variables were sequentially removed based on the highest p-value until only those with independent and statistically significant associations with hospital mortality remained (Page 15, Lines 331-341). This approach ensured a balanced, evidence-based model grounded in both statistical rigor and clinical insight.

If not already done, confirm that you checked for multicollinearity between predictors and that the events-per-variable ratio was sufficient for a reliable model. Citing a sample size rule (e.g., requiring ~50 events per variable) could reinforce that the analysis is well-powered

Our answer:

Thank you for your insightful comment. To ensure the robustness of our model, we evaluated multicollinearity using the Variance Inflation Factor (VIF) for each predictor included in the final model (Page 15, Lines 342–344). All VIF values were below the commonly accepted threshold of 5, indicating that multicollinearity was not a concern and supporting the stability and interpretability of the regression coefficients. Additional details are provided in the note box beneath Table 8 and under S14 Table within the S1 File. We also adhered to the recommended events-per-variable (EPV) ratio, ensuring that the number of outcome events (deaths) was sufficient to support the number of predictors included (Page 15, Lines 343–345). This helped mitigate the risk of overfitting and enhanced the generalizability of our findings. Model performance was further evaluated using multiple diagnostic criteria, including the Hosmer-Lemeshow goodness-of-fit test, pseudo-R^2 values, and log-likelihood

statistics (Page 15, Lines 345–347). These metrics confirmed acceptable model calibration and discrimination. Additional details are provided in the note box beneath Table 8 and under S14 Table within the S1 File. We appreciate your guidance in strengthening the methodological clarity of our study and believe these revisions reinforce the rigor and reliability of our analysis.

In the Discussion, consider highlighting how your results align with prior studies. For example, earlier research has shown that delayed intubation in ARDS leads to higher mortality (56% vs 36% when intubation is early) This supports your finding that patients intubated during transfer had better outcomes. Drawing this parallel and citing such studies will strengthen the rationale for your recommendation on early intubation. Likewise, the classic risk factors you identified (age, severity scores, shock) are well known in ARDS nature, acknowledging this shows that your data are consistent with existing knowledge.

Our answer:

Thank you for your valuable and constructive feedback. We have revised the Discussion section to contextualize our findings within the existing body of literature, as you suggested. These refinements clarify the clinical implications of our findings and further substantiate the rationale for improving pre-hospital airway management in resource-limited settings. The revised text now reads:

"Patient transfers from local to central hospitals may further worsen outcomes. Most patients in our study were transferred without ET or MV (Table 1, S1 Table as shown in S1 File), and a low rate of ET use during transport was independently associated with increased hospital mortality (Table 8, S14 Table in S1 File). A German prospective cohort study demonstrated that mechanically ventilated patients with ARDS experienced fewer transport-related complications, highlighting the importance of optimized inter-hospital transfer protocols.(55) Similarly, a United States observational study found that delayed intubation was associated with higher mortality rates (50.0% [18/36] vs. 29.6% [104/351] for early intubation and 14.3% [10/70] for patients never requiring intubation).(22) These findings suggest that early interventions, such as high-flow nasal cannula (HFNC) therapy, non-invasive ventilation, or intubation, may influence outcomes.(22, 56) Although our study lacks data on HFNC or non-

invasive MV during transport, prior research in Vietnam identifies suboptimal pre- and inter-hospital care as a risk factor for poor outcomes(57). Improving patient transfer services, including access to critical interventions (e.g., HFNC, intubation, MV) and optimized ambulance systems, is essential." (Pages 31-32, Lines 532-546)

The observed in-hospital mortality of 61.5% is quite high. It would be valuable to discuss possible reasons and compare with international figures. For instance, a large international ARDS study reported ~40% mortality; the substantially higher rate in your cohort may reflect the severity of cases referred to your center or resource limitations (e.g., fewer ICU interventions like ECMO). Emphasize how this finding underlines the urgency of improving critical care capacity. You might suggest specific strategies, such as better training for pre-hospital emergency teams in airway management or ensuring ventilators and skilled personnel are available during transport, to reduce delays in optimal care.

Our answer:

Thank you very much for your insightful and constructive feedback. In response, we have substantially revised the Discussion section to address the high hospital mortality rate (61.5%) observed in our ARDS cohort. We now contextualize this figure by comparing it with international data, including the ~40% mortality reported in a large multicenter ARDS study. This comparison underscores the potential impact of factors such as the severity of cases referred to our center and limitations in critical care resources. To further strengthen the discussion, we emphasize the urgent need to expand critical care capacity in resource-constrained settings. We have incorporated specific, actionable strategies, such as enhanced training for pre-hospital emergency teams in airway management and ensuring the availability of ventilators and skilled personnel during patient transport, to help mitigate delays in initiating optimal care. These points are now integrated throughout the revised Discussion section to highlight both the challenges and potential solutions. (Pages 30-35, Lines 505-629)

Since you report that 36.7% of patients underwent cytokine adsorption therapy, readers may wonder if this intervention had any impact on outcomes. If data permit, consider commenting on whether therapies like cytokine adsorption (or others such as prone positioning, ECMO if applicable) were associated with improved outcomes or not. Even if a formal analysis wasn't done, a short note acknowledging this point would be useful – for example, clarifying that cytokine adsorption was a rescue therapy used in the sickest patients (hence its effect is hard to discern) or that its benefit could not be confirmed in this study. This would address a potential question in readers' minds about the role of such treatments.

Our answer:

Thank you for your insightful and constructive suggestion. We agree that acknowledging the potential impact of cytokine adsorption therapy (ECAT), even without a formal efficacy analysis, adds valuable context for readers. In response, we have revised the Discussion section to include a brief commentary on the role of ECAT in ARDS management. Specifically, we note that ECAT was primarily employed as a rescue therapy in the most critically ill patients, making it difficult to isolate its effect on outcomes. We also clarify that, within the scope of our study, no definitive benefit of ECAT could be established. This addition aims to address potential questions regarding the therapeutic relevance of ECAT and similar interventions such as prone positioning and ECMO. The revised text now reads:

"ARDS is increasingly recognized as a syndrome of systemic immune dysregulation. Although the concept of a cytokine storm was initially suggested,(76) later studies have indicated that systemic cytokine levels in ARDS patients are generally lower than previously thought, except in severe COVID-19 cases(26, 77). The use of ECAT has been explored as a potential intervention for immune modulation. While detailed data on ECAT were not available in this study, the use of ECAT was associated with an increased risk of hospital mortality (S14 Table as shown in S1 File), likely reflecting its role as a rescue therapy for critically ill patients (S15 Table as shown in S1 File). This pattern suggests confounding by indication rather than a direct harmful effect. Given the observational nature of our study, definitive conclusions regarding the efficacy or safety of ECAT must be validated through prospective studies. Similarly, adjunctive therapies such as prone positioning and ECMO, which are typically reserved for patients with refractory hypoxemia (S16 Table as shown in

S1 File), warrant further investigation better to understand their impact on outcomes in ARDS patients." (Page 34, Lines 591-603)

Before final publication, a careful read-through for minor language edits would be helpful. The manuscript is generally well-written, so only small tweaks are needed – e.g., adding missing words ("were") or articles, and maintaining consistent tense. Ensuring each abbreviation is defined at first use and used consistently (for example, using either "ET" or "endotracheal tube" uniformly after definition) will avoid any confusion. These changes are minor but will polish the text and improve readability for a broad audience.

Our answer:

We sincerely thank the Reviewer for the constructive feedback. In response, we have carefully revised the manuscript to address all suggested language improvements. Specifically:

- We conducted a thorough read-through to correct minor grammatical issues, including the addition of missing words and articles, and ensured consistent verb tense throughout the text.

- All abbreviations have been reviewed to ensure they are clearly defined at first mention and used consistently thereafter (e.g., "endotracheal tube (ET)" is now used uniformly throughout the manuscript).

- We made minor stylistic and syntactic edits to enhance clarity and readability for a broad audience.

We appreciate the Reviewer's attention to detail, which has helped us further refine the manuscript.

By addressing the above points, the manuscript's clarity and impact will be further enhanced. The core content and conclusions are strong; these suggestions are intended to refine the presentation and provide additional context for readers.

Our answer:

We sincerely thank the Reviewer for the thoughtful and encouraging feedback. We have carefully addressed all the suggested points to enhance the clarity and presentation of the manuscript. We genuinely appreciate the Reviewer's insights, which have helped us strengthen the manuscript's clarity and impact for a broader readership.

Thank the Editors and Reviewers so much for taking the time to leave their high-quality reviews.

Sincerely,

Chinh Quoc Luong, MD, PhD
Neuro Intensive Care Department,
Neurology Center, Bach Mai Hospital,
No. 78, Giai Phong, Dong Da district,
Hanoi 100000, Vietnam
Email: luongquocchinh@gmail.com

Son Ngoc Do, MD, PhD
Center for Critical Care Medicine,
Bach Mai Hospital,
No. 78, Giai Phong Road, Dong Da District,
Hanoi, 100000, Vietnam.
Email: sonngocdo@gmail.com
