# Supplementary material for: Factors related to mortality in patients with acute respiratory distress syndrome (ARDS) in a lower middle-income country: A retrospective observational study
Source: PLoS One. 2025 Nov 18;20(11):e0337071. doi: 10.1371/journal.pone.0337071 (PMC12626306; doi:10.1371/journal.pone.0337071)
Supplement: Attachment — Submitted filename: Response to Reviewers.docx [file pone.0337071.s005.docx]

**Dr. Gurmeet Singh, M.D., Ph.D.**

Academic Editor

PLOS ONE

*November 3, 2025*

Dear Dr. Gurmeet Singh,

On behalf of all the authors, we are resubmitting our revised manuscript titled *“Factors related to mortality in patients with acute respiratory distress syndrome (ARDS) in a lower middle-income country: a retrospective observational study”* (PONE-D-24-35750R2).

We sincerely appreciate the thoughtful comments and suggestions provided by the Editors and Reviewers. We have carefully addressed all feedback and revised our manuscript accordingly. These insights have significantly improved the quality of our work, and we hope the Editor finds our revised manuscript suitable for publication in PLOS ONE.

We confirm that this research is original, has not been published elsewhere, and is not currently under consideration by any other journal. All authors have read and approved the manuscript, have agreed on the order of authorship, and hold the necessary permissions and rights for the data presented.

Below, we have included our point-by-point responses to the comments from the Editor and the Reviewers. Thank you for considering this submission.

Sincerely,

**Chinh Quoc Luong, MD, PhD**

Neuro Intensive Care Department,

Neurology Center, Bach Mai Hospital,

No. 78, Giai Phong, Dong Da district,

Hanoi 100000, Vietnam

Email: luongquocchinh@gmail.com

**Son Ngoc Do, MD, PhD**

Center for Critical Care Medicine,

Bach Mai Hospital,

No. 78, Giai Phong Road, Dong Da District,

Hanoi, 100000, Vietnam.

Email: sonngocdo@gmail.com

*****

We thank the Editors and the Reviewers for the valuable comments and suggestions that have helped us improve the contents of this paper. In what follows, we have used the **boldface** to indicate the Editors' and the Reviewers' comments and the standard font face for our responses. We also highlighted in yellow the modifications that we performed on the manuscript.

**RESPONSE TO EDITOR COMMENTS**

**PONE-D-24-35750R2**

**Factors related to mortality in patients with acute respiratory distress syndrome (ARDS) in a lower middle-income country: a retrospective observational study**

**PLOS ONE**

**Dear Dr. Do,**

**Thank you for submitting your manuscript to PLOS ONE. After careful consideration, we feel that it has merit but does not fully meet PLOS ONE’s publication criteria as it currently stands. Therefore, we invite you to submit a revised version of the manuscript that addresses the points raised during the review process.**

Our answer:

Thank you for your encouraging feedback and for the opportunity to revise our manuscript.

We sincerely appreciate the time and effort that you and the reviewers have devoted to evaluating our work. In response to the thoughtful comments and suggestions provided, we have carefully revised the manuscript to address all points raised during the review process. These changes have strengthened the clarity, rigor, and overall quality of our study.

We look forward to your continued evaluation and hope the revised version meets the journal’s publication criteria.

**ACADEMIC EDITOR:**

**Thank you for submitting the revised version of your manuscript entitled "Factors related to mortality in patients with acute respiratory distress syndrome (ARDS) in a lower middle-income country: a retrospective observational study" to PLOS ONE. We appreciate the substantial effort you have made to address the previous reviewer and editorial comments. Both reviewers acknowledge that the manuscript has improved considerably, with clearer presentation, stronger methodology, and an extended dataset that enhances the study’s relevance. The focus on inter-hospital transport and pre-hospital care adds an important dimension that is often underrepresented in the ARDS literature, particularly in resource-limited settings.**

**After careful consideration of the reviewers’ feedback, the editorial decision is: Minor Revision.**

Our answer:

Thank you for your thoughtful and encouraging feedback regarding our revised manuscript.

We are grateful to you and the reviewers for recognizing the improvements in clarity, methodology, and dataset relevance. We appreciate your acknowledgment of the added focus on inter-hospital transport and pre-hospital care, which makes a meaningful contribution to the literature on acute respiratory distress syndrome (ARDS) in resource-limited settings.

We will carefully address the remaining points raised and submit the minor revision promptly. Thank you again for your continued guidance and support throughout the review process.

**While the manuscript is close to being suitable for publication, several refinements are still recommended to strengthen the clarity and impact of your work:**

**Mortality Rate Contextualization**

**Please provide a deeper discussion of the high observed mortality rate (61.5%), particularly in relation to referral bias, resource limitations, and the availability of advanced therapies.**

Our answer:

We sincerely thank the Academic Editor for this important suggestion. In the revised manuscript, we have expanded the discussion to provide deeper contextualization of the high mortality rate. Specifically, we have now highlighted the potential impact of referral bias resulting from the transfer of more severe cases from local hospitals, the limitations in critical care resources at both the local and central levels, and the restricted availability of advanced therapies, such as ECMO and high-flow nasal cannula (HFNC). We have explicitly discussed these factors to better explain the observed mortality rate in comparison with international cohorts, as follows:

“A key factor in our study is the selective nature of our cohort, as most ARDS patients were transferred from local hospitals to a central tertiary facility (Table 1), introducing significant referral bias. Patients are typically transferred only after initial management fails at lower-level facilities, resulting in a cohort with more advanced disease severity upon arrival. In Vietnam, patients with ARDS are often initially diagnosed with severe pneumonia at local hospitals and transferred only when their condition worsens, delaying diagnosis and treatment and potentially contributing to higher mortality (26, 27, 31, 32). Resource limitations further compound this issue. Vietnam's healthcare system faces chronic underfunding, shortages of trained intensivists, advanced diagnostic equipment, and critical care beds, particularly at provincial and district levels (levels II and III per the Ministry of Health, (29). These constraints lead to suboptimal early interventions, such as delayed initiation of lung-protective ventilation or adjunctive therapies, which are more readily available in HICs.

Pneumonia was the predominant etiology of ARDS in our study (Table 3), aligning with prior studies such as VALID (19.3%; 125/646, (8), ALIEN (42.4%; 108/255, (6), and LUNG SAFE (59.4%; 1794/3022, (9), though our cohort exhibited a higher prevalence. This predominance likely exacerbates mortality, as pneumonia and sepsis are leading causes of early death in ARDS, alongside MODS (12-15). In the present study, the SOFA score (Table 4) is comparable to those in LUNG SAFE (mean 10.1 [95% CI: 9.9–10.2], (9), but our PaO2/FiO2 ratio is lower (Table 4 vs. 161 mmHg [95% CI: 158–163] in LUNG SAFE, (9), suggesting greater hypoxemia severity. A large observational study (n=3670) also indicates that mortality rises with ARDS severity (19, 36), potentially explaining our elevated mortality rates due to the high pneumonia burden. Additionally, the lack of widespread access to advanced therapies, such as ECMO (utilized in only 8.5% of our cohort; Table 5) and HFNC during transport or early management (29, 30, 55), limits rescue options for refractory cases. In contrast to HICs, where ECMO is more routinely available and associated with better outcomes in high-volume centers (56, 57), LMICs like Vietnam face barriers including high costs, limited expertise, infrastructure gaps, and global disparities in access. These factors collectively contribute to the higher mortality observed here compared to international cohorts (e.g., 23.7% in VALID (8) and 40.1% in LUNG SAFE (9)). To reduce ARDS mortality in Vietnam, enhancing local healthcare resources (e.g., human expertise, medical equipment, and infrastructure) is critical, as evidenced by ongoing efforts to upgrade hospitals and address workforce shortages.” (Lines 519-550, Pages31-32)

**COVID-19 Subgroup Analysis**

**Expand the discussion to more explicitly address how the pandemic may have shaped ARDS case-mix, transport practices, and access to ventilatory support.**

Our answer:

We thank the Academic Editor for this valuable recommendation. We have revised the discussion to more explicitly address how the COVID-19 pandemic influenced ARDS case-mix, transport practices, and access to ventilatory support. We now describe how infection control measures, resource constraints, and evolving treatment protocols during the pandemic altered referral patterns, increased the use of endotracheal intubation during transport, and reduced the availability of invasive ventilation at local hospitals. These contextual factors are now integrated into the interpretation of our COVID-19 subgroup findings, as follows:

“During the pandemic, the ARDS case-mix shifted significantly, with a marked decline in cases attributed to seasonal viruses, such as influenza A (H1N1; Table 7). This decline was due to non-pharmaceutical interventions (NPIs) such as mask-wearing and social distancing (87), mirroring global trends (87, 88), which contrasts with pre-pandemic data in Vietnam, where influenza was a primary cause of ARDS, underscoring the need for robust epidemiological surveillance to monitor such changes. Conversely, there was an influx of ARDS cases associated with SARS-CoV-2, particularly the Delta variant, as critically ill COVID-19 patients often faced delayed hospital admissions, leading to severe pneumonia, rapid ARDS progression, and high mortality (25, 78). This etiological shift resulted in a more homogeneous viral-driven ARDS cohort during the pandemic, which differed from the diverse pre-pandemic mix of bacterial and other viral pneumonias, and potentially influenced treatment protocols in resource-limited settings. There was also a significant reduction in the use of both noninvasive and invasive MV at referring hospitals (Table 7). This decline stemmed from resource constraints (29), including acute ventilator shortages in LMICs that restricted MV access, and infection control measures to limit aerosol-generating procedures, aligning with reports of strained healthcare systems worldwide (78, 88, 89). Such limitations exacerbated challenges in early respiratory support, prompting greater reliance on alternatives, such as HFNC, when available (90). Transport practices evolved as reliance on ET use increased during inter-hospital transfers to tertiary centers (Table 7), reflecting adaptations to manage infectious risks, stabilize airways in deteriorating patients, and cope with overwhelmed local facilities amid surges. This trend, observed in other LMICs (91, 92), often led to increased transfers for critical cases due to uneven resource distribution. Still, it also added logistical and psychological burdens on patients and providers (93, 94). Vietnam's shortages of ventilators and critical care infrastructure were key drivers of these changes. A notable shift in ARDS management was the reduced use of invasive MV on the first day of admission during the pandemic (Table 7). This change is consistent with evolving guidelines favoring noninvasive strategies, such as HFNC, for COVID-19-related ARDS to conserve ventilators and minimize intubation risks (57, 90). Pre-pandemic, invasive MV was standard (19, 36); this change arose from both resource constraints and emerging evidence (57), underscoring the need for context-specific protocols in low-resource settings. Despite these shifts, hospital mortality rates for ARDS remained stable between pre-pandemic and pandemic periods (Table 7), suggesting resilience in Vietnam's critical care system. However, rates were consistently high, exceeding the 35–45% reported in HICs (9), likely due to limited infrastructure, including shortages of trained intensivists, ventilators, and standardized protocols.” (Lines 650-684, Pages36-37)

**Transport Data Limitations**

**Highlight the limitations of the transport-related data more clearly, and if possible, provide a conceptual framework to guide interpretation of these findings.**

Our answer:

We appreciate this thoughtful comment. In the revised limitations section, we have expanded our discussion of the transport data, noting the incomplete documentation of transfer duration, level of care provided en route, and vehicle type. To guide interpretation, we have added a conceptual framework that emphasizes how deficiencies in pre-hospital and inter-hospital systems—such as a lack of standardization, limited monitoring, and staffing variability—may have influenced outcomes. This framework helps contextualize the observed association between endotracheal intubation during transport and reduced mortality. Further details as follows:

“This study has several limitations that warrant careful consideration when interpreting the findings. First, the retrospective design limited data availability and completeness across key variables (S17 Table in S1 File). Transport-related information was available for only 214 patients and lacked critical details, such as transfer duration, en route care level, vehicle type, and interventions. These gaps introduce potential selection bias into the multivariable logistic regression analyses, as unmeasured confounders, such as transfer delays or variations in monitoring, could affect patients' arrival condition and hospital mortality, which undermines model robustness, potentially leading to over- or under-estimation of associations and unreliable inferences about transport's role in ARDS outcomes. Vietnam's pre- and inter-hospital transfer systems remain underdeveloped, lacking standardization, integration, and regulatory oversight (30, 32, 34, 55, 95), which hampers data collection for surveillance, quality improvement, and research and limits the generalizability of our findings to other LMICs with similar constraints. To interpret these transport limitations, we adapt Donabedian's healthcare quality framework, categorizing factors into structural, process, and outcome dimensions, thereby highlighting how deficiencies in LMIC inter-hospital transport for ARDS patients amplify risks, unlike in HICs with specialized retrieval teams and protocols. Structural quality (i) involves foundational resources, such as ambulance types (hospital-operated vs. private/informal), staffing (trained physicians vs. nurses/bystanders), and equipment (ventilators, oxygen, ECMO). Incomplete documentation in our study underscores LMIC challenges, such as resource scarcity, potentially underestimating risks from equipment failure or inadequate stabilization. Process quality (ii) covers protocols and actions, including pre-transfer stabilization, communication, and interventions like ET or MV. Our data showed limited ET use (60.1% of transported patients), possibly delaying respiratory support and worsening hypoxemia; non-standardized processes may bias toward poorer outcomes. Outcome quality (iii) includes transport events (e.g., hypoxia, hypotension) and metrics like hospital mortality. ET use during transport was independently associated with lower mortality (Table 8; S14 Table in S1 File), but missing details hinder causal inferences due to unadjusted confounders. Despite these issues, our findings suggest prioritizing ET could mitigate transfer risks. Future prospective studies should use standardized protocols, including mobile ECMO or ET/MV interventions, to optimize ARDS transport in LMICs.” (Lines 685-715, Pages 37-38)

**Adjunctive Therapies**

**A brief discussion on adjunctive therapies (e.g., prone positioning, ECMO, HFNC, cytokine adsorption) would strengthen completeness, even if data remain limited.**

Our answer:

We thank the Academic Editor for this excellent suggestion. We have added a dedicated section in the discussion addressing adjunctive therapies, including cytokine adsorption, prone positioning, ECMO, and HFNC. While acknowledging the limited data available, we now provide context on their use in our cohort, including their potential benefits and risks, as well as how their application in resource-limited settings may differ from that in high-income countries. This addition enhances the comprehensiveness of our discussion and situates our findings within the broader context of ARDS management literature. Further details as follows:

“ARDS is increasingly recognized as a syndrome of systemic immune dysregulation. Although the concept of a cytokine storm was initially proposed (77), later studies have shown that systemic cytokine levels in ARDS patients are generally lower than previously thought, except in severe COVID-19 cases (26, 78). The use of ECAT has been explored for immune modulation. In our study, detailed ECAT data were limited. Still, its application was associated with higher hospital mortality (S14 Table in S1 File), likely because it was used as rescue therapy in critically ill patients (S15 Table in S1 File), suggesting confounding by indication rather than direct harm. A systematic review of hemadsorption (a form of ECAT) in ARDS patients on veno-venous ECMO highlighted potential benefits, including reduced inflammation and improved oxygenation (79). However, the evidence is confined to small cohorts with limited long-term mortality data. A meta-analysis further supported the role of adjunctive hemadsorption in improving clinical outcomes in ARDS, but called for larger trials (80). In resource-limited settings, such as Vietnam, where ECAT is reserved for severe cases due to cost and availability constraints, LMIC-focused studies are needed to evaluate its cost-effectiveness and integration with standard care. Prone positioning, utilized in nearly a quarter of our cohort (Table 5), primarily for patients with severe hypoxemia (S16 Table in S1 File). This approach aligns with established guidelines for moderate-to-severe ARDS, as it improves ventilation-perfusion matching and reduces ventilator-induced lung injury (57). A narrative review on biomarkers in COVID-19 and non-COVID-19 ARDS supports their role in reducing mortality in severe cases, particularly when used early and for prolonged periods (81). A systematic review and meta-analysis demonstrated that combining prone positioning with ECMO improves short-term survival in severe ARDS, with greater benefits in non-COVID patients (82). However, barriers in our setting, such as staffing shortages and pandemic-related infection control measures, may have limited wider use, underscoring the need for LMIC-specific training programs. ECMO was applied to a notable subset of our patients (Table 5), exceeding the rate in the LUNG SAFE study (3.2%; 76/2377, (9), possibly reflecting higher disease severity or better local access. A review of ECMO in severe ARDS emphasizes its role in stabilizing refractory hypoxemia (83). Still, it stresses careful patient selection and multidisciplinary management to address amplified complications, such as bleeding and infection, in resource-limited environments. A review of hypoxemic respiratory failure in cardiac patients further highlights these challenges (84). Although HFNC was not explicitly detailed in our dataset (potentially under oxygen supplements in Table 5), it is increasingly supported as a non-invasive option for mild-to-moderate ARDS to delay intubation. A multicenter prospective cohort study showed that early prone positioning with HFNC or noninvasive ventilation reduced intubation rates by up to 50% in patients with moderate-to-severe ARDS, improving oxygenation and comfort (85). Recent studies reinforce the efficacy of prone positioning in awake HFNC patients for COVID-19 ARDS, reducing MV needs (86). In Vietnam, amid ventilator shortages, HFNC could address early management gaps, but prospective LMIC trials are essential to assess feasibility and outcomes.” (Lines 610-649, Pages 34-36)

**Language and Style**

**We encourage further polishing of the language, including shortening long sentences, ensuring abbreviation consistency, and improving overall readability.**

**Please revise the manuscript accordingly and submit the revised version together with a detailed response letter indicating how each point has been addressed.**

Our answer:

We appreciate this helpful feedback. We have carefully revised the entire manuscript to enhance readability by condensing overly long sentences, ensuring consistent use of abbreviations (e.g., standardizing terms like ARDS, ECMO, HFNC, and MV throughout), and refining the language for clarity and flow. These changes were applied globally, with particular attention to the Abstract, Methods, Results, and Discussion sections to improve conciseness and coherence without altering the scientific content. For example, complex sentences in the Discussion were broken into shorter ones, and abbreviations were defined upon first use and used consistently thereafter. The marked-up manuscript highlights these refinements in yellow.

**Please submit your revised manuscript by Nov 14 2025 11:59PM. If you will need more time than this to complete your revisions, please reply to this message or contact the journal office at plosone@plos.org. When you're ready to submit your revision, log on to https://www.editorialmanager.com/pone/ and select the 'Submissions Needing Revision' folder to locate your manuscript file.**

Our answer:

Thank you for providing the submission deadline and clear instructions for uploading our revised manuscript.

We confirm that the revised manuscript has now been completed and submitted through the Editorial Manager system by November 14, 2025, as requested. We appreciate your continued guidance and support throughout the review process, and we look forward to the next steps in the editorial evaluation.

**Please include the following items when submitting your revised manuscript:**

**▪ A rebuttal letter that responds to each point raised by the academic editor and reviewer(s). You should upload this letter as a separate file labeled 'Response to Reviewers'.**

**▪ A marked-up copy of your manuscript that highlights changes made to the original version. You should upload this as a separate file labeled 'Revised Manuscript with Track Changes'.**

**▪ An unmarked version of your revised paper without tracked changes. You should upload this as a separate file labeled 'Manuscript'.**

Our answer:

Thank you very much for your guidance and for outlining the requirements for our revised manuscript submission.

We confirm that we have included all the requested items:

- A rebuttal letter addressing each point raised by the academic editor and reviewers, uploaded as 'Response to Reviewers'.

- A marked-up version of the manuscript with tracked changes, uploaded as 'Revised Manuscript with Track Changes'.

- A clean version of the revised manuscript without tracked changes, uploaded as 'Manuscript'.

We appreciate your support and look forward to the next steps in the review process.

**If you would like to make changes to your financial disclosure, please include your updated statement in your cover letter. Guidelines for resubmitting your figure files are available below the reviewer comments at the end of this letter.**

Our answer:

Thank you for your guidance regarding the financial disclosure and resubmission of the figure file.

We have carefully reviewed our financial disclosure and confirm that no changes are necessary at this time. The original statement remains accurate and has been retained in our revised submission.

We also appreciate the clarification provided on figure file resubmission and have followed the outlined guidelines to ensure full compliance with the journal’s requirements.

**If applicable, we recommend that you deposit your laboratory protocols in protocols.io to enhance the reproducibility of your results. Protocols.io assigns your protocol its own identifier (DOI) so that it can be cited independently in the future. For instructions see: https://journals.plos.org/plosone/s/submission-guidelines#loc-laboratory-protocols. Additionally, PLOS ONE offers an option for publishing peer-reviewed Lab Protocol articles, which describe protocols hosted on protocols.io. Read more information on sharing protocols at https://plos.org/protocols?utm_medium=editorial-email&utm_source=authorletters&utm_campaign=protocols.**

Our answer:

Thank you for the helpful suggestion regarding the use of protocols.io to enhance reproducibility.

We fully support the initiative to promote transparency by sharing detailed protocols. However, our study did not involve laboratory-based procedures and therefore does not include laboratory protocols applicable for deposition. Instead, we conducted a retrospective observational study and comprehensively described the corresponding study protocol in the Methods section of our revised manuscript.

We hope this clarifies our methodological framework. Please let us know if any further documentation would be helpful.

**We look forward to receiving your revised manuscript.**

**Kind regards,**

**Gurmeet Singh, M.D., Ph.D.,**

**Academic Editor**

**PLOS ONE**

Our answer:

We sincerely thank you and the reviewers for your thoughtful and constructive feedback. We truly appreciate the time and effort invested in evaluating our manuscript and providing high-quality reviews. Your insights have been invaluable in helping us enhance the clarity and overall quality of our work.

We have carefully addressed all comments and incorporated the necessary revisions. We look forward to the continued editorial evaluation of our revised submission.

**Journal Requirements:**

**1. If the reviewer comments include a recommendation to cite specific previously published works, please review and evaluate these publications to determine whether they are relevant and should be cited. There is no requirement to cite these works unless the editor has indicated otherwise.**

Our answer:

Thank you for the clarification regarding citation of previously published works.

We confirm that the reviewer comments did not include any recommendations to cite specific references. As such, no additional citations were necessary in our revised manuscript.

We appreciate your continued guidance throughout the editorial process.

**2. Please review your reference list to ensure that it is complete and correct. If you have cited papers that have been retracted, please include the rationale for doing so in the manuscript text, or remove these references and replace them with relevant current references. Any changes to the reference list should be mentioned in the rebuttal letter that accompanies your revised manuscript. If you need to cite a retracted article, indicate the article’s retracted status in the References list and also include a citation and full reference for the retraction notice.**

Our answer:

Thank you for your guidance regarding the reference list.

We have thoroughly reviewed all cited works and confirm that none of the references included in our manuscript have been retracted. Therefore, no changes to the reference list were necessary in this revision.

We appreciate your continued support throughout the editorial process.

**RESPONSE TO REVIEWER COMMENTS**

**Reviewers' comments:**

**Reviewer's Responses to Questions**

**Comments to the Author**

**1. If the authors have adequately addressed your comments raised in a previous round of review and you feel that this manuscript is now acceptable for publication, you may indicate that here to bypass the “Comments to the Author” section, enter your conflict of interest statement in the “Confidential to Editor” section, and submit your "Accept" recommendation.**

**Reviewer #2: All comments have been addressed**

**Reviewer #3: All comments have been addressed**

Our answer:

We sincerely thank you for your thoughtful evaluation and positive feedback on our revised manuscript.

We are grateful for your time and effort in reviewing our work across multiple rounds, and we genuinely appreciate your acknowledgment that all comments have been adequately addressed. Your insights have been instrumental in helping us improve the clarity, rigor, and relevance of our study.

Thank you again for your valuable contributions to the review process.

**2. Is the manuscript technically sound, and do the data support the conclusions?**

**The manuscript must describe a technically sound piece of scientific research with data that supports the conclusions. Experiments must have been conducted rigorously, with appropriate controls, replication, and sample sizes. The conclusions must be drawn appropriately based on the data presented.**

**Reviewer #2: Yes**

**Reviewer #3: Yes**

Our answer:

Thank you for your positive evaluation regarding the technical soundness of our manuscript and the alignment of our data with the study’s conclusions.

We are grateful for your recognition of the rigor and methodological integrity of our research. Your feedback affirms the strength of our approach and the relevance of our findings, and we genuinely appreciate your support throughout the review process.

**3. Has the statistical analysis been performed appropriately and rigorously?**

**Reviewer #2: Yes**

**Reviewer #3: Yes**

Our answer:

Thank you for your positive assessment regarding the statistical analysis in our manuscript.

We are grateful for your recognition of the rigor and appropriateness of our analytical approach. Your feedback confirms the validity of our methods and enhances overall confidence in our findings.

We appreciate your time and thoughtful evaluation throughout the review process.

**4. Have the authors made all data underlying the findings in their manuscript fully available?**

**The PLOS Data policy requires authors to make all data underlying the findings described in their manuscript fully available without restriction, with rare exception (please refer to the Data Availability Statement in the manuscript PDF file). The data should be provided as part of the manuscript or its supporting information, or deposited to a public repository. For example, in addition to summary statistics, the data points behind means, medians and variance measures should be available. If there are restrictions on publicly sharing data—e.g. participant privacy or use of data from a third party—those must be specified.**

**Reviewer #2: Yes**

**Reviewer #3: Yes**

Our answer:

Thank you for your positive assessment regarding our data availability.

We appreciate your recognition that all data underlying the findings in our manuscript have been made fully accessible in accordance with PLOS ONE’s data policy. We have ensured that the relevant datasets are included in the manuscript and supporting information, with appropriate documentation to facilitate transparency and reproducibility.

Thank you again for your thoughtful review and support.

**5. Is the manuscript presented in an intelligible fashion and written in standard English?**

**PLOS ONE does not copyedit accepted manuscripts, so the language in submitted articles must be clear, correct, and unambiguous. Any typographical or grammatical errors should be corrected at revision, so please note any specific errors here.**

**Reviewer #2: Yes**

**Reviewer #3: Yes**

Our answer:

Thank you for your positive feedback regarding the clarity and language quality of our manuscript.

We appreciate your recognition that the manuscript is presented in an intelligible manner and written in standard English. We have taken care to ensure that the text is clear, correct, and free of typographical or grammatical errors in accordance with the journal’s guidelines.

Thank you again for your thoughtful review and support.

**6. Review Comments to the Author**

**Please use the space provided to explain your answers to the questions above. You may also include additional comments for the author, including concerns about dual publication, research ethics, or publication ethics. (Please upload your review as an attachment if it exceeds 20,000 characters)**

**Reviewer #2: You guys did a solid job with the revisions. The paper is clearer, more rigorous, and adds useful insights into ARDS outcomes in a resource-limited setting. The focus on transport and pre-hospital care makes it stand out, since most ARDS studies focus only on ICU interventions.**

**Main Points:**

**They addressed all prior reviewer/editor concerns. The issue of “dual publication” is clarified—this is an updated dataset with a new focus, not a duplicate.**

**You added a COVID vs. pre-COVID analysis, which showed no major difference in mortality but gives extra context.**

**The stats section is much stronger now. You explained how they built the model, checked for multicollinearity (all fine), and discussed missing data honestly.**

**Key result: intubation during transport strongly predicts survival. The effect size is big, but given the wide confidence intervals, I’d interpret it cautiously. Still, it’s a meaningful and practice-relevant finding.**

**You added discussion of cytokine adsorption therapy—helpful for context, while noting it was used mainly in very sick patients.**

Our answer:

Thank you very much for your encouraging and detailed feedback on our revised manuscript.

We truly appreciate your recognition of the improvements made in clarity, methodological rigor, and the added insights into ARDS outcomes in resource-limited settings. Your comments affirm the value of highlighting transport and pre-hospital care, an often-overlooked aspect in ARDS research, and we’re grateful that this dimension stood out to you.

We’re also pleased that the clarification regarding dataset updates and the inclusion of the COVID-19 vs. pre-COVID-19 analysis provided meaningful context. Your acknowledgment of the strengthened statistical section, including our approach to model building, multicollinearity checks, and transparency around missing data, is deeply appreciated.

Regarding the key finding on intubation during transport, we agree with your interpretation and have noted the wide confidence intervals in our limitations section to encourage cautious but constructive consideration of its clinical relevance, as follows:

“Finally, the sample size (353 patients, 21 covariates) met traditional events-per-variable thresholds of 10–20 (51, 52) but falls short of newer recommendations (e.g., 50, requiring at least 500 patients; (53, 54). Wide confidence intervals for ET's protective effect necessitate caution. Further studies with larger sample sizes and more diverse populations will be essential to validate and expand upon these findings, enhancing their reliability and relevance.” (Lines 721-726, Page 39)

We also appreciate your positive remarks on the inclusion of cytokine adsorption therapy, which has added important nuance to the treatment landscape.

Thank you again for your thoughtful review and support throughout this process.

**Reviewer #3: The manuscript has been substantially improved compared to the earlier version, and the authors have clearly made an effort to address the reviewers’ and editor’s concerns. The extended dataset, inclusion of the pandemic period, and focus on inter-hospital transport strengthen the study and add relevance. The finding regarding endotracheal intubation during transfer remains a novel and clinically meaningful contribution.**

Our answer:

Thank you very much for your thoughtful and encouraging feedback on our revised manuscript.

We truly appreciate your recognition of the improvements made and your acknowledgment of our efforts to address the concerns of the reviewers and the editor. We are pleased that the extended dataset, inclusion of the pandemic period, and emphasis on inter-hospital transport have added relevance and strengthened the study.

Your positive remarks regarding the findings on endotracheal intubation during transfer are especially appreciated. We agree that this represents a novel and clinically meaningful contribution, and we have aimed to present it in an appropriate context and with caution.

Thank you again for your valuable insights and support throughout the review process.

**Some areas would still benefit from refinement:**

**- The high mortality rate (61.5%) needs deeper contextualization, particularly in relation to referral bias, resource limitations, and lack of advanced therapies.**

Our answer:

We sincerely thank the reviewer for this important suggestion. In the revised manuscript, we have expanded the discussion to provide deeper contextualization of the high mortality rate. Specifically, we have now highlighted the potential impact of referral bias resulting from the transfer of more severe cases from local hospitals, the limitations in critical care resources at both the local and central levels, and the restricted availability of advanced therapies, such as ECMO and high-flow nasal cannula (HFNC). We have explicitly discussed these factors to better explain the observed mortality rate in comparison with international cohorts, as follows:

“A key factor in our study is the selective nature of our cohort, as most ARDS patients were transferred from local hospitals to a central tertiary facility (Table 1), introducing significant referral bias. Patients are typically transferred only after initial management fails at lower-level facilities, resulting in a cohort with more advanced disease severity upon arrival. In Vietnam, patients with ARDS are often initially diagnosed with severe pneumonia at local hospitals and transferred only when their condition worsens, delaying diagnosis and treatment and potentially contributing to higher mortality (26, 27, 31, 32). Resource limitations further compound this issue. Vietnam's healthcare system faces chronic underfunding, shortages of trained intensivists, advanced diagnostic equipment, and critical care beds, particularly at provincial and district levels (levels II and III per the Ministry of Health, (29). These constraints lead to suboptimal early interventions, such as delayed initiation of lung-protective ventilation or adjunctive therapies, which are more readily available in HICs.

Pneumonia was the predominant etiology of ARDS in our study (Table 3), aligning with prior studies such as VALID (19.3%; 125/646, (8), ALIEN (42.4%; 108/255, (6), and LUNG SAFE (59.4%; 1794/3022, (9), though our cohort exhibited a higher prevalence. This predominance likely exacerbates mortality, as pneumonia and sepsis are leading causes of early death in ARDS, alongside MODS (12-15). In the present study, the SOFA score (Table 4) is comparable to those in LUNG SAFE (mean 10.1 [95% CI: 9.9–10.2], (9), but our PaO2/FiO2 ratio is lower (Table 4 vs. 161 mmHg [95% CI: 158–163] in LUNG SAFE, (9), suggesting greater hypoxemia severity. A large observational study (n=3670) also indicates that mortality rises with ARDS severity (19, 36), potentially explaining our elevated mortality rates due to the high pneumonia burden. Additionally, the lack of widespread access to advanced therapies, such as ECMO (utilized in only 8.5% of our cohort; Table 5) and HFNC during transport or early management (29, 30, 55), limits rescue options for refractory cases. In contrast to HICs, where ECMO is more routinely available and associated with better outcomes in high-volume centers (56, 57), LMICs like Vietnam face barriers including high costs, limited expertise, infrastructure gaps, and global disparities in access. These factors collectively contribute to the higher mortality observed here compared to international cohorts (e.g., 23.7% in VALID (8) and 40.1% in LUNG SAFE (9)). To reduce ARDS mortality in Vietnam, enhancing local healthcare resources (e.g., human expertise, medical equipment, and infrastructure) is critical, as evidenced by ongoing efforts to upgrade hospitals and address workforce shortages.” (Lines 519-550, Pages31-32)

**- The COVID-19 subgroup analysis is useful, but the discussion could emphasize more explicitly how the pandemic may have shaped ARDS case-mix, transport practices, and access to ventilatory support.**

Our answer:

We thank the reviewer for this valuable recommendation. We have revised the discussion to more explicitly address how the COVID-19 pandemic influenced ARDS case-mix, transport practices, and access to ventilatory support. We now describe how infection control measures, resource constraints, and evolving treatment protocols during the pandemic altered referral patterns, increased the use of endotracheal intubation during transport, and reduced the availability of invasive ventilation at local hospitals. These contextual factors are now integrated into the interpretation of our COVID-19 subgroup findings, as follows:

“During the pandemic, the ARDS case-mix shifted significantly, with a marked decline in cases attributed to seasonal viruses, such as influenza A (H1N1; Table 7). This decline was due to non-pharmaceutical interventions (NPIs) such as mask-wearing and social distancing (87), mirroring global trends (87, 88), which contrasts with pre-pandemic data in Vietnam, where influenza was a primary cause of ARDS, underscoring the need for robust epidemiological surveillance to monitor such changes. Conversely, there was an influx of ARDS cases associated with SARS-CoV-2, particularly the Delta variant, as critically ill COVID-19 patients often faced delayed hospital admissions, leading to severe pneumonia, rapid ARDS progression, and high mortality (25, 78). This etiological shift resulted in a more homogeneous viral-driven ARDS cohort during the pandemic, which differed from the diverse pre-pandemic mix of bacterial and other viral pneumonias, and potentially influenced treatment protocols in resource-limited settings. There was also a significant reduction in the use of both noninvasive and invasive MV at referring hospitals (Table 7). This decline stemmed from resource constraints (29), including acute ventilator shortages in LMICs that restricted MV access, and infection control measures to limit aerosol-generating procedures, aligning with reports of strained healthcare systems worldwide (78, 88, 89). Such limitations exacerbated challenges in early respiratory support, prompting greater reliance on alternatives, such as HFNC, when available (90). Transport practices evolved as reliance on ET use increased during inter-hospital transfers to tertiary centers (Table 7), reflecting adaptations to manage infectious risks, stabilize airways in deteriorating patients, and cope with overwhelmed local facilities amid surges. This trend, observed in other LMICs (91, 92), often led to increased transfers for critical cases due to uneven resource distribution. Still, it also added logistical and psychological burdens on patients and providers (93, 94). Vietnam's shortages of ventilators and critical care infrastructure were key drivers of these changes. A notable shift in ARDS management was the reduced use of invasive MV on the first day of admission during the pandemic (Table 7). This change is consistent with evolving guidelines favoring noninvasive strategies, such as HFNC, for COVID-19-related ARDS to conserve ventilators and minimize intubation risks (57, 90). Pre-pandemic, invasive MV was standard (19, 36); this change arose from both resource constraints and emerging evidence (57), underscoring the need for context-specific protocols in low-resource settings. Despite these shifts, hospital mortality rates for ARDS remained stable between pre-pandemic and pandemic periods (Table 7), suggesting resilience in Vietnam's critical care system. However, rates were consistently high, exceeding the 35–45% reported in HICs (9), likely due to limited infrastructure, including shortages of trained intensivists, ventilators, and standardized protocols.” (Lines 650-684, Pages36-37)

**- The limitations regarding transport data should be highlighted further; if possible, provide a conceptual framework to guide interpretation.**

Our answer:

We appreciate this thoughtful comment. In the revised limitations section, we have expanded our discussion of the transport data, noting the incomplete documentation of transfer duration, level of care provided en route, and vehicle type. To guide interpretation, we have added a conceptual framework that emphasizes how deficiencies in pre-hospital and inter-hospital systems—such as a lack of standardization, limited monitoring, and staffing variability—may have influenced outcomes. This framework helps contextualize the observed association between endotracheal intubation during transport and reduced mortality. Further details as follows:

“This study has several limitations that warrant careful consideration when interpreting the findings. First, the retrospective design limited data availability and completeness across key variables (S17 Table in S1 File). Transport-related information was available for only 214 patients and lacked critical details, such as transfer duration, en route care level, vehicle type, and interventions. These gaps introduce potential selection bias into the multivariable logistic regression analyses, as unmeasured confounders, such as transfer delays or variations in monitoring, could affect patients' arrival condition and hospital mortality, which undermines model robustness, potentially leading to over- or under-estimation of associations and unreliable inferences about transport's role in ARDS outcomes. Vietnam's pre- and inter-hospital transfer systems remain underdeveloped, lacking standardization, integration, and regulatory oversight (30, 32, 34, 55, 95), which hampers data collection for surveillance, quality improvement, and research and limits the generalizability of our findings to other LMICs with similar constraints. To interpret these transport limitations, we adapt Donabedian's healthcare quality framework, categorizing factors into structural, process, and outcome dimensions, thereby highlighting how deficiencies in LMIC inter-hospital transport for ARDS patients amplify risks, unlike in HICs with specialized retrieval teams and protocols. Structural quality (i) involves foundational resources, such as ambulance types (hospital-operated vs. private/informal), staffing (trained physicians vs. nurses/bystanders), and equipment (ventilators, oxygen, ECMO). Incomplete documentation in our study underscores LMIC challenges, such as resource scarcity, potentially underestimating risks from equipment failure or inadequate stabilization. Process quality (ii) covers protocols and actions, including pre-transfer stabilization, communication, and interventions like ET or MV. Our data showed limited ET use (60.1% of transported patients), possibly delaying respiratory support and worsening hypoxemia; non-standardized processes may bias toward poorer outcomes. Outcome quality (iii) includes transport events (e.g., hypoxia, hypotension) and metrics like hospital mortality. ET use during transport was independently associated with lower mortality (Table 8; S14 Table in S1 File), but missing details hinder causal inferences due to unadjusted confounders. Despite these issues, our findings suggest prioritizing ET could mitigate transfer risks. Future prospective studies should use standardized protocols, including mobile ECMO or ET/MV interventions, to optimize ARDS transport in LMICs.” (Lines 685-715, Pages 37-38)

**- A short discussion on adjunctive therapies (cytokine adsorption, prone positioning, ECMO, HFNC) would strengthen the completeness of the discussion, even if data are limited.**

Our answer:

We thank the reviewer for this excellent suggestion. We have added a dedicated section in the discussion addressing adjunctive therapies, including cytokine adsorption, prone positioning, ECMO, and HFNC. While acknowledging the limited data available, we now provide context on their use in our cohort, including their potential benefits and risks, as well as how their application in resource-limited settings may differ from that in high-income countries. This addition enhances the comprehensiveness of our discussion and situates our findings within the broader context of ARDS management literature. Further details as follows:

“ARDS is increasingly recognized as a syndrome of systemic immune dysregulation. Although the concept of a cytokine storm was initially proposed (77), later studies have shown that systemic cytokine levels in ARDS patients are generally lower than previously thought, except in severe COVID-19 cases (26, 78). The use of ECAT has been explored for immune modulation. In our study, detailed ECAT data were limited. Still, its application was associated with higher hospital mortality (S14 Table in S1 File), likely because it was used as rescue therapy in critically ill patients (S15 Table in S1 File), suggesting confounding by indication rather than direct harm. A systematic review of hemadsorption (a form of ECAT) in ARDS patients on veno-venous ECMO highlighted potential benefits, including reduced inflammation and improved oxygenation (79). However, the evidence is confined to small cohorts with limited long-term mortality data. A meta-analysis further supported the role of adjunctive hemadsorption in improving clinical outcomes in ARDS, but called for larger trials (80). In resource-limited settings, such as Vietnam, where ECAT is reserved for severe cases due to cost and availability constraints, LMIC-focused studies are needed to evaluate its cost-effectiveness and integration with standard care. Prone positioning, utilized in nearly a quarter of our cohort (Table 5), primarily for patients with severe hypoxemia (S16 Table in S1 File). This approach aligns with established guidelines for moderate-to-severe ARDS, as it improves ventilation-perfusion matching and reduces ventilator-induced lung injury (57). A narrative review on biomarkers in COVID-19 and non-COVID-19 ARDS supports their role in reducing mortality in severe cases, particularly when used early and for prolonged periods (81). A systematic review and meta-analysis demonstrated that combining prone positioning with ECMO improves short-term survival in severe ARDS, with greater benefits in non-COVID patients (82). However, barriers in our setting, such as staffing shortages and pandemic-related infection control measures, may have limited wider use, underscoring the need for LMIC-specific training programs. ECMO was applied to a notable subset of our patients (Table 5), exceeding the rate in the LUNG SAFE study (3.2%; 76/2377, (9), possibly reflecting higher disease severity or better local access. A review of ECMO in severe ARDS emphasizes its role in stabilizing refractory hypoxemia (83). Still, it stresses careful patient selection and multidisciplinary management to address amplified complications, such as bleeding and infection, in resource-limited environments. A review of hypoxemic respiratory failure in cardiac patients further highlights these challenges (84). Although HFNC was not explicitly detailed in our dataset (potentially under oxygen supplements in Table 5), it is increasingly supported as a non-invasive option for mild-to-moderate ARDS to delay intubation. A multicenter prospective cohort study showed that early prone positioning with HFNC or noninvasive ventilation reduced intubation rates by up to 50% in patients with moderate-to-severe ARDS, improving oxygenation and comfort (85). Recent studies reinforce the efficacy of prone positioning in awake HFNC patients for COVID-19 ARDS, reducing MV needs (86). In Vietnam, amid ventilator shortages, HFNC could address early management gaps, but prospective LMIC trials are essential to assess feasibility and outcomes.” (Lines 610-649, Pages 34-36)

**- Minor polishing of language (shorter sentences, abbreviation consistency) would improve readability.**

Our answer:

We appreciate this helpful feedback. We have carefully revised the manuscript to enhance readability by condensing overly long sentences, ensuring consistent use of abbreviations, and refining the language throughout. These refinements have improved the clarity and flow of the text.

**Overall, this revision represents a meaningful contribution to the ARDS literature in resource-limited settings. With the suggested refinements, it should be suitable for publication.**

Our answer:

Thank you very much for your encouraging feedback and thoughtful evaluation of our revised manuscript.

We are grateful for your recognition of the study’s contribution to the literature on acute respiratory distress syndrome (ARDS), particularly in resource-limited settings. Your support affirms the relevance of our findings and the value of the refinements made during the revision process.

We appreciate your time and expertise, and we look forward to the possibility of publication.

**7. PLOS authors have the option to publish the peer review history of their article (what does this mean?). If published, this will include your full peer review and any attached files.**

**If you choose “no”, your identity will remain anonymous but your review may still be made public.**

**Do you want your identity to be public for this peer review? For information about this choice, including consent withdrawal, please see our Privacy Policy.**

**Reviewer #2: Yes: Sagar Bathija**

**Reviewer #3: No**

Our answer:

Thank you for indicating your preferences regarding the publication of your peer review identities.

We sincerely appreciate your thoughtful evaluations and constructive feedback throughout the review process. We respect your individual choices and are grateful for your contributions, whether made anonymously or publicly.

Thank the Editors and Reviewers so much for taking the time to leave their high-quality reviews.

Sincerely,

**Chinh Quoc Luong, MD, PhD**

Neuro Intensive Care Department,

Neurology Center, Bach Mai Hospital,

No. 78, Giai Phong, Dong Da district,

Hanoi 100000, Vietnam

Email: luongquocchinh@gmail.com

**Son Ngoc Do, MD, PhD**

Center for Critical Care Medicine,

Bach Mai Hospital,

No. 78, Giai Phong Road, Dong Da District,

Hanoi, 100000, Vietnam.

Email: sonngocdo@gmail.com
